# LPS Induces mTORC1 and mTORC2 Activation During Monocyte Adhesion

**DOI:** 10.3389/fmolb.2018.00067

**Published:** 2018-07-18

**Authors:** Marcelle C. Ribeiro, Diogo B. Peruchetti, Leandro S. Silva, João L. Silva-Filho, Mariana C. Souza, Maria das Graças Henriques, Celso Caruso-Neves, Ana Acacia S. Pinheiro

**Affiliations:** ^1^Instituto de Biofísica Carlos Chagas Filho, Universidade Federal do Rio de Janeiro, Rio de Janeiro, Brazil; ^2^Fundação Oswaldo Cruz, Instituto de Tecnologia em Fármacos, Rio de Janeiro, Brazil; ^3^Instituto Nacional de Ciência e Tecnologia em Medicina Regenerativa, Rio de Janeiro, Brazil

**Keywords:** lipopolysaccharide, mTORC1, mTORC2, protein kinase C, phorbol ester, human acute monocytic leukemia cell line, monocyte, adhesion

## Abstract

Monocyte adhesion is a crucial step in transmigration and can be induced by lipopolysaccharide (LPS). Here, we studied the role of mammalian target of rapamycin (mTOR) complexes, mTORC1 and mTORC2, and PKC in this process. We used THP-1 cells, a human monocytic cell line, to investigate monocyte adhesion under static and flow conditions. We observed that 1.0 μg/mL LPS increased PI3K/mTORC2 pathway and PKC activity after 1 h of incubation. WYE-354 10^−6^ M (mTORC2/mTORC1 inhibitor) and 10^−6^ M wortmannin avoided monocyte adhesion in culture plates. In addition, WYE also blocked LPS-induced CD11a expression. Interestingly, rapamycin and WYE-354 blocked both LPS-induced monocyte adhesion in a cell monolayer and actin cytoskeleton rearrangement, confirming mTORC1 involvement in this process. Once activated, PKC activates mTORC1/S6K pathway in a similar effect observed to LPS. Activation of the mTORC1/S6K pathway was attenuated by 10^−6^ M U0126, an MEK/ERK inhibitor, and 10^−6^ M calphostin C, a PKC inhibitor, indicating that the MEK/ERK/TSC2 axis acts as a mediator. In agreement, 80 nM PMA (a PKC activator) mimicked the effect of LPS on the activation of the MEK/ERK/TSC2/mTORC1/S6K pathway, monocyte adhesion to ECV cells and actin cytoskeleton rearrangement. Our findings show that LPS induces activation of mTOR complexes. This signaling pathway led to integrin expression and cytoskeleton rearrangement resulting in monocyte adhesion. These results describe a new molecular mechanism involved in monocyte adhesion in immune-based diseases.

## Introduction

Acute and chronic inflammatory responses are characterized by the recruitment of circulating monocytes to inflamed tissues and further differentiation into macrophage or myeloid dendritic cells (Ingersoll et al., 2011). The emigration of monocytes from blood involves sequential molecular interactions with endothelial cells, known as the adhesion cascade, in which firm adhesion to the endothelium is a fundamental step (Imhof and Aurrand-Lions, 2004). Monocyte integrin activation and adhesion molecule expression in the endothelium are induced by several factors, such as lipopolysaccharide (LPS), through activation of different signaling pathways (Hmama et al., 1999; Kounalakis and Corbett, 2006; Xu et al., 2013; Lee et al., 2014). LPS, an outer membrane component of gram-negative bacteria, directly activates monocytes, leading to its adherence in endothelial cells or the extracellular matrix (Hmama et al., 1999; Kounalakis and Corbett, 2006). Monocyte adhesion induced by 1 μg/mL LPS follows a transient profile and depends on integrin expression and activation, including CD11a and CD11b (Hmama et al., 1999; Kounalakis and Corbett, 2006; Lee et al., 2014). However, the molecular mechanism behind the effect of LPS on monocyte adhesion is still poorly understood. Therefore, exploration of the signaling pathways involved in monocyte adhesion is important to understand the pathophysiology of several immune-based diseases.

It is known that the effects of LPS are mediated by Toll-like receptor 4 (TLR4), the activation of which triggers different protein kinases, including protein kinase C (PKC) isoforms (Roman et al., 2004; Fronhofer et al., 2006; Zhou et al., 2006). It has been shown that PKC is involved in actin remodeling and integrin expression/activation in leukocytes (Abram and Lowell, 2009; Fogh et al., 2014). Specifically, in human monocytes, Roman et al. (2004) demonstrated that β1 integrin expression, induced by LPS, was PKC dependent. Furthermore, Mine et al. (2002) reported the involvement of PKC on monocyte adhesion to endothelium induced by oxidized low density lipoprotein (oxLDL). Accordingly, it was shown that phorbol esters, such as PMA (extensively used as a PKC activator), induces monocyte adhesion and differentiation into macrophages (Tsuchiya et al., 1982; Chang et al., 2012).

To become activated, all PKC isoforms need to be phosphorylated in at least three different motifs: the turn motif, the hydrophobic motif, and the activation loop (Hage-Sleiman et al., 2015). The first two motifs are phosphorylated by mammalian target of rapamycin (mTOR) complex 2 (mTORC2) (Ikenoue et al., 2008). mTOR is a serine/threonine protein kinase (catalytic unit) that forms two complexes in mammalian cells: (1) mTORC1 associated with regulatory-associated protein of TOR (RAPTOR), and (2) mTORC2 associated with rapamycin-insensitive companion of TOR (RICTOR) (Laplante and Sabatini, 2012; Peruchetti et al., 2014; Shimobayashi and Hall, 2014). Usually, mTORC2 leads to the activation of mTORC1 (Peruchetti et al., 2014; Shimobayashi and Hall, 2014). Some evidence highlights the possible involvement of mTORC1 and mTORC2 on actin cytoskeleton rearrangement, one characteristic of adhered monocytes (Jacinto et al., 2004; Liu et al., 2008; Chen et al., 2015). Jacinto et al. (2004) showed that silencing an mTORC2-specific regulatory protein abolished F-actin polarization in HEK cells. In addition, it has been shown that rapamycin (an mTOR inhibitor) prevents both cell adhesion and F-actin reorganization induced by insulin-like growth factor 1 (IGF-1) in different tumor cell lines (Liu et al., 2008; Chen et al., 2015). However, the exact role of mTORC1 and mTORC2 in monocyte adhesion has never been reported and requires further exploration.

Here, we used THP-1 cells (a human acute monocytic leukemia cell line) to evaluate the role of mTORC1 and mTORC2 in monocyte adhesion following LPS stimuli. We observed that both mTORC1 and mTORC2 complexes participate in LPS-induced monocyte adhesion under static and flow conditions. In this process, LPS induced PKC activation in a phosphatidylinositol 3-kinase (PI3K)/mTORC2-dependent manner. In turn, PKC activation leads to ERK/mTORC1/S6K pathway activation. Disturbance of this signaling cascade impaired the expression of CD11a as well as actin polymerization and focal adhesion formation. These results unravel a new molecular mechanism behind monocyte adhesion and, consequently, the transmigration process during pathological events in which monocytes migration plays a crucial role, such as in acute and chronic diseases.

## Methods

### Materials and reagents

LPS (055: B5), HEPES, tris, phosphate-buffered saline (PBS), sodium bicarbonate, phenylmethane sulfonyl fluoride (PMSF), sodium orthovanadate, sodium fluoride, sodium β-glycerophosphate, sodium pyrophosphate, sodium azide, protease inhibitor, adenosine triphosphate (ATP), magnesium chloride, EDTA, histone type II-S, calphostin C and rapamycin were acquired from Sigma-Aldrich (St. Louis, M. O., USA). DMSO was acquired from Merck-Chemicals (Merck, Darmstadt, Germany). PMA, wortmannin and WYE-354 were purchased from EMD Millipore (Billerica, M. A., USA). U0126 and all antibodies used for immunoblotting, except anti-phospho-TSC2, were purchased by Cell Signaling Technology (Danvers, M. A., USA). The monoclonal anti-tuberin (phospho S664) antibody was purchased from Abcam (Cambridge, M. A., USA). The ECL Prime Western Blotting Detection Reagent was acquired from GE Healthcare Life Sciences (Little Chalfont, Buckinghamshire, UK). Anti-CD11a fluorescein isothiocyanate (FITC)-conjugated and anti-CD11b phosphoethanolamine (PE)-conjugated monoclonal antibodies were obtained from BD Pharmingen (San Diego, C. A., USA). DAPI, 4′,6-diamidino-2-phenylindole, was purchased from Life Technologies (Carlsbad, C. A., USA). RPMI 1640, fetal bovine serum (FBS), penicillin/streptomycin and trypan blue were acquired from GIBCO (Grand Island, N. Y., USA). ^32^Pi was obtained from Instituto Brasileiro de Pesquisas Energéticas e Nucleares (São Paulo, S. P., Brazil) and [γ-^32^P] ATP was prepared according to Maia et al. (1993). All other reagents were of the highest purity available.

### Cell culture

THP-1 cells, a human acute monocytic leukemia cell line, and ECV-304 cells, a human bladder carcinoma T24 cell line (Dirks et al., 1999; Brown et al., 2000), traditionally used as model to study adhesion processes (Böger et al., 2000; Souza et al., 2012; Hien et al., 2014), were kindly provided by Dr. Maria das Graças Henriques (Fiocruz, R. J., Brazil). THP-1 cells were cultivated in RPMI 1640 medium supplemented with 10% FBS and 1% penicillin/streptomycin at 37°C/5% CO_2_. For immunoblotting and flow cytometry analysis, 2 × 10^6^ THP-1 cells were seeded in a 6-well plate the day before each experiment. ECV-304 cells were cultured in RPMI 1640 medium supplemented with 10% FBS, 2 mM l-glutamine and 20 mg/L gentamicin. ECV-304 cells (5 × 10^4^ cells) were seeded in 24-well culture plates for 24 h or until they reached < 50% confluence to perform static adhesion experiments. In order to perform underflow adhesion experiments, ECV-304 cells were plated in 35 × 10 mm dishes, and the experiments were accomplished using at least 90% cell confluence.

### Adhesion assay under static conditions

To evaluate monocyte adhesion under static conditions, we performed two adhesion assays: (1) using uncoated culture plates, and (2) using ECV-304 cells. Although ECV-304 cells are not endothelial cells, they are usually used for adhesion studies because they shares the expression of intercellular cell adhesion molecule-1 (ICAM-1) with human cells (Lidington et al., 1999). For adhesion assay to culture plates, 1 μg/mL LPS or 80 nM PMA was added to a THP-1 cell suspension and incubated for different times. When required, cells were pretreated with inhibitors for 30 min before the adhesion assay. After incubation, cells that remained adhered to the culture plate were removed with cold 0.5 mM PBS-EDTA, centrifuged, and stored until quantification. The supernatant containing non-adhered cells was also harvested, centrifuged, and stored. Cell quantification was realized in an automatic counter (Bio-Rad TC-20), considering only viable cells by trypan blue exclusion. In all assays, the cell viability was >95%. The percentage adhesion represents the number of adhered cells of the total number of cells (adherent + non-adherent). For the monocyte adhesion assay to ECV cells, 4 × 10^5^ THP-1 cells were pretreated with 10^−9^ M rapamycin or 10^−6^ M WYE-354 for 15 min followed by treatment with 1 μg/mL LPS or 80 nM PMA. After 15 min, THP-1 cells were washed with 1 × PBS and incubated with ECV cells for 1 h at 37°C with agitation. After this time, non-adhered cells were removed by washing with 1 × PBS and adhered monocytes were stained by Diff-Quick. Images were obtained by light microscopy and adherence was quantified by analyzing 10 random fields. The adhesion index was calculated according to Souza et al. (2012): adhesion index = {[(ECV with bound monocytes)/total number of ECV] × [(monocytes attached to ECV)/total number of ECV]} × 100.

### Adhesion assay under flow

THP-1 cells (3 × 10^5^ cells/mL) stimulated with 1 μg/mL LPS or 80 nM PMA were pretreated with 10^−9^ M rapamycin or 10^−6^ M WYE-354 and resuspended in RPMI 1640 medium with 20% v/v Percoll (GE Healthcare) to increase the density of the medium and avoid cell sedimentation. Then, THP-1 cells were perfused over the ECV-304 monolayers through a syringe pump (PHD2000, Harvard Apparatus Inc., Holliston, MA) for 1 h at a mean flow rate of 1.6 mL/min. The flow was then interrupted and the dish containing both ECV cells and monocytes was stained by Diff-Quick. Images of adherent THP-1 cells on the monolayers were obtained using light microscopy in 10 random selected microscope fields. Results are expressed as number of adhered monocytes per field.

### Protein kinase C activity assay

The specific activity of PKC was measured by the difference in ^32^P incorporated from [γ-^32^P]ATP to histone protein in the presence and absence of calphostin C (10^−8^ M), a specific enzyme inhibitor, as determined previously (Arnaud-Batista et al., 2016). The reaction was initiated by the addition of 10 μM ATP (specific activity × 7 μCi/μmol [γ-^32^P]ATP), to a reaction medium containing 4 mM MgCl_2_, 20 mM HEPES-Tris (pH 7.0), 1.5 mg/mL histone, and 0.7 mg/mL cell lysate. The reaction took place at 37°C and was stopped after 20 min by adding 30% trichloroacetic acid (TCA) and immediately placed on ice until filtration. Filtration was performed adding 200 μL of sample through a Millipore filter (0.45 mM) on suction. The filters were washed with an ice-cold solution of 20% TCA and then 2 mM phosphate buffer (pH 7.0). The radioactivity incorporated into the membrane was quantified by liquid scintillation counting (Packard Tri-Carb 2100 TR).

### Immunoblotting assay

The immunoblotting assay was performed as shown previously (Peruchetti et al., 2011, 2014; Arnaud-Batista et al., 2016). Briefly, after treatment, non-adherent and adherent cells were washed 3 times with 1 × PBS, collected and incubated in lysis buffer (20 mM HEPES [pH 7.4], 2 mM EGTA, 1% Triton X-100, 50 mM sodium fluoride, 5 mM sodium orthovanadate, 5 mM sodium pyrophosphate, 10 mM sodium β-glycerophosphate, 1 mM PMSF and 2 × protease inhibitor cocktail) for 40 min. The cells were then clarified by centrifugation (4°C for 13 min at 15,000 × g). The supernatant was collected and the concentration of protein was quantified by the Bradford method (Bradford, 1976). The proteins were resolved on sodium dodecyl sulfate 9% acrylamide gels and transferred to a polyvinylidene fluoride membrane (Millipore). After incubation with specific primary and secondary antibodies, detection of proteins of interest was carried out by adding ECL plus as substrate. Images were acquired by chemiluminescence (Image Quant LAS4000; GE Healthcare Life Sciences). All acquired images were processed by adjusting the brightness and contrast using NIH ImageJ software (version 1.6.0). This image processing method was applied to every pixel in the original image without changing the information illustrated. The intensity of the bands was also quantified using ImageJ. Original images are avaliable as Supplementary Files (Data Sheets 1–4).

### Phosphorylation of mTOR, ERK1/2 and TSC2

Phosphorylation of mTOR, ERK1/2 and TSC2 was quantified by immunoblotting as described previously (Peruchetti et al., 2014; Arnaud-Batista et al., 2016). Specific antibodies used for the detection of S2481 and S2448 phosphorylated residues on mTOR were rabbit polyclonal S2481 phospho-mTOR (no. 2974; dilution 1:1,000) and monoclonal S2448 phospho-mTOR (clone D9C2; no. 5536; dilution 1:1,000) antibodies. Total mTOR was detected using rabbit monoclonal mTOR antibody (clone 7C10; no. 2983; dilution 1:1,000). ERK phosphorylation was detected using monoclonal antibody against Thr-202/Tyr-204 phosphorylated residue on ERK (no. 9101S, dilution 1:1,000), and total ERK (monoclonal anti-ERK1/2; no. 9102S; dilution 1:1,000). The monoclonal antibody against phosphorylated Ser-664 on TSC2 (no. ab133465; dilution 1:1,000) and monoclonal antibody against total TSC2 (clone D93F12; no. 4308S; dilution 1:1,000) was used to detect TSC2 phosphorylation. In each experiment, the phospho bands were normalized to total protein bands obtained after stripping and re-probing the same membrane with the corresponding antibodies.

### mTORC1 and mTORC2 activity assay

mTORC1 and mTORC2 activity was determined through immunoblotting as described previously (Peruchetti et al., 2014; Arnaud-Batista et al., 2016). Briefly, mTORC2 activity was realized by detecting Akt/PKB phosphorylation using a rabbit polyclonal phospho-Akt/PKB (Ser-473) antibody (no. 9271; dilution 1:1,000) and normalized to total protein using rabbit polyclonal Akt/PKB (no. 9272; dilution: 1:1,000). In addition, mTORC1 activity was realized by detecting S6K phosphorylation using rabbit monoclonal S6K (Thr-389) (clone 108D2; no. 9234; dilution 1:1,000) antibody and normalized to total S6K using S6K antibody (no. 9202; dilution 1:1,000).

### Flow cytometry

After treatment, THP-1 monocytes were incubated for 30 min at 4°C in RPMI-10% FBS and 0.1% sodium azide to block non-specific binding sites. Then, the cells were washed and incubated with anti-human CD11a antibody conjugated to FITC (0.5 μg/mL) or anti-human CD11b antibody conjugated to PE (1 μg/mL) for 30 min at 4°C in RPMI-10% FBS and 0.1% sodium azide. Analysis of surface cells markers was performed using FlowJo software (version 7.6.2). All results were obtained using CellQuest software on a FACScan (Becton Dickinson). In each sample, at least 10^5^ cells were used for incubation with respective antibodies. All data are presented as histograms on a log scale of fluorescence intensity.

### Filamentous actin staining

Filamentous actin staining was determined as described previously (Silva-Filho et al., 2013). PMA- or LPS-stimulated monocytes (5 × 10^3^) pretreated with 10^−9^ M rapamycin or 10^−6^ M WYE-354 were resuspended in 100 μL of RPMI 1640 plus 1% bovine serum albumin (BSA). These cells were allowed to adhere for 1 h to coverslips coated with fibronectin (10 μg/mL). Thereafter, the plate was washed 3 times with 1 × PBS and adhered cells were fixed with 4% paraformaldehyde (v/v) in 1 × PBS (pH 7.0) at 24°C. Fixed cells were first permeabilized for 40 min with 3% NP-40 at 4°C, and subsequently for 15 min with acetone at −20°C. Then, for the next 20 min, monocytes were quenched using a solution of 50 mM ammonium chloride and 3% BSA in 1 × PBS. Cells were incubated with 0.4 units of Alexa Fluor 546- phalloidin (Invitrogen, Eugene, O. R., USA) in methanol in a humidified chamber for 1 h at 4°C. Finally, the cells were quenched in 3% BSA/PBS for 20 min and mounted in Entellan medium. Cells were examined with a fluorescence microscope (Eclipse 80i, Nikon, Japan), with 40 × objective. The images were acquired with QCapture software (version 2.0.13) and analyzed with NIH ImageJ software (version 1.6.0).

### Statistical analysis

The results are expressed as means ± standard error of at least three independent experiments. GraphPad Prism 5 (version 5.01, GraphPad Software, San Diego California, U. S. A., www.graphpad.com) was used for statistical analysis. Differences between groups were compared by one-way analysis of variance (ANOVA), followed by the Newman-Keuls post-test. The assumption of normality and homoscedasticity were checked by Shapiro-Wilk test and Leven's test, respectively. Significance was determined as *P* < 0.05.

## Results

### LPS induces monocyte adhesion through the PI3K/PKC pathway

**Figure 1:**
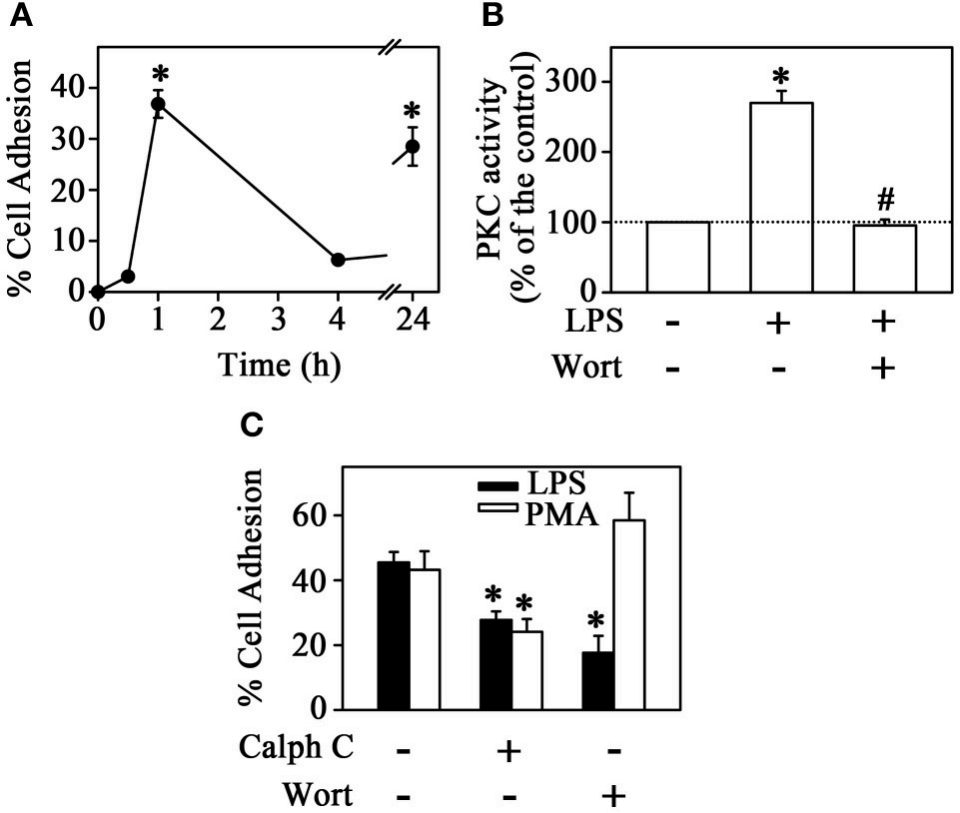
PKC mediates LPS-induced monocyte adhesion. **(A)** THP-1 cells were incubated with 1 μg**/**mL LPS for different times and culture plate adhesion was measured (*n* = 4). **(B)** Effect of LPS on PKC activity in the presence or absence of 10^−6^ M Wortmannin (*n* = 5). **(C)** The effect of 10^−6^ M calphostin C and 10^−6^ M Wortmannin on LPS- or PMA-induced THP-1 cell adhesion (*n* = 3). PKC, protein kinase C; Calph C, calphostin C; Wort, wortmannin. The results are presented as means ± SE. *p < 0.05 vs. unstimulated non-adhered cells (control); #*p* < 0.05 vs. LPS.

Initially, we tested the effect of LPS on the induction of monocyte adhesion. For this, THP-1 cells were incubated with 1.0 μg/mL LPS for 0.5, 1, 4, or 24 h and the percentage of adhered cells on the culture plate was determined. Figure 1A shows that cell adhesion followed a transient profile reaching a maximum effect at 1 h and returning to control levels after 4 h of incubation. However, after longer periods of incubation (24 h), adhesion recovered to 30%, as previously described by Kounalakis and Corbett (2006). Attachment induced by 80 nM PMA followed a different profile, in which the percentage of adhesion increased linearly with time and most cells adhered to the tissue culture dishes after 24 h incubation (data not shown). This adhesion profile has been reported and it is associated to cell differentiation triggered by PMA (Chang et al., 2012).

Multiple signaling mechanisms exist to regulate adhesion/migration of different cell types. The PI3K and PKC signaling pathways are key regulators of this process induced by chemokines or cytokines (Fogh et al., 2014; Filippi, 2016; Dwyer et al., 2017). However, the effect of LPS on the induction of this mechanisms is still poorly understood. In the next step, we tested the possible effect of LPS on PKC activity by the histone phosphorylation method. Figure 1B shows that LPS induced a 2.7-fold increase in PKC activity after 1 h incubation. The increase in PKC activity correlates with the increased cell adhesion induced by LPS. Accordingly, at 1 h incubation, PMA (used as a PKC activator) increased cell adhesion in a similar way (Figure 1C). Furthermore, pre-treatment with 10^−6^ M calphostin C, a PKC competitive inhibitor, prevented the stimulatory effect of both LPS and PMA on cell adhesion (Figure 1C). To evaluate whether the activation of PKC promoted by LPS is dependent on PI3K activity (Yuan and Guan, 2015), THP-1 cells were treated with 10^−6^ M wortmannin (a PI3K inhibitor) before incubation with LPS or PMA. Under these conditions, adhesion was reduced to around 50%, as observed earlier with calphostin C treatment alone. However, the addition of wortmannin inhibited LPS-induced adhesion specifically and did not change the effect of PMA (Figure 1C). Moreover, in this experimental condition, the stimulatory effect of LPS on PKC activity was totally abolished (Figure 1B). These results suggest that LPS produced a stimulatory effect on monocyte adhesion by activating PKC in a PI3K-dependent manner.

### mTORC2 mediates PKC activation induced by LPS

A vast literature has shown that mTOR complexes are central in governing cell growth, proliferation, autophagy and survival (Laplante and Sabatini, 2012; Shimobayashi and Hall, 2014). More recently, the importance of such complexes in regulating cell adhesion has been pointed out and, in this context, mTORC2 regulates adhesion via an Akt-independent mechanism (Chen et al., 2015; Sato et al., 2016).

**Figure 2:**
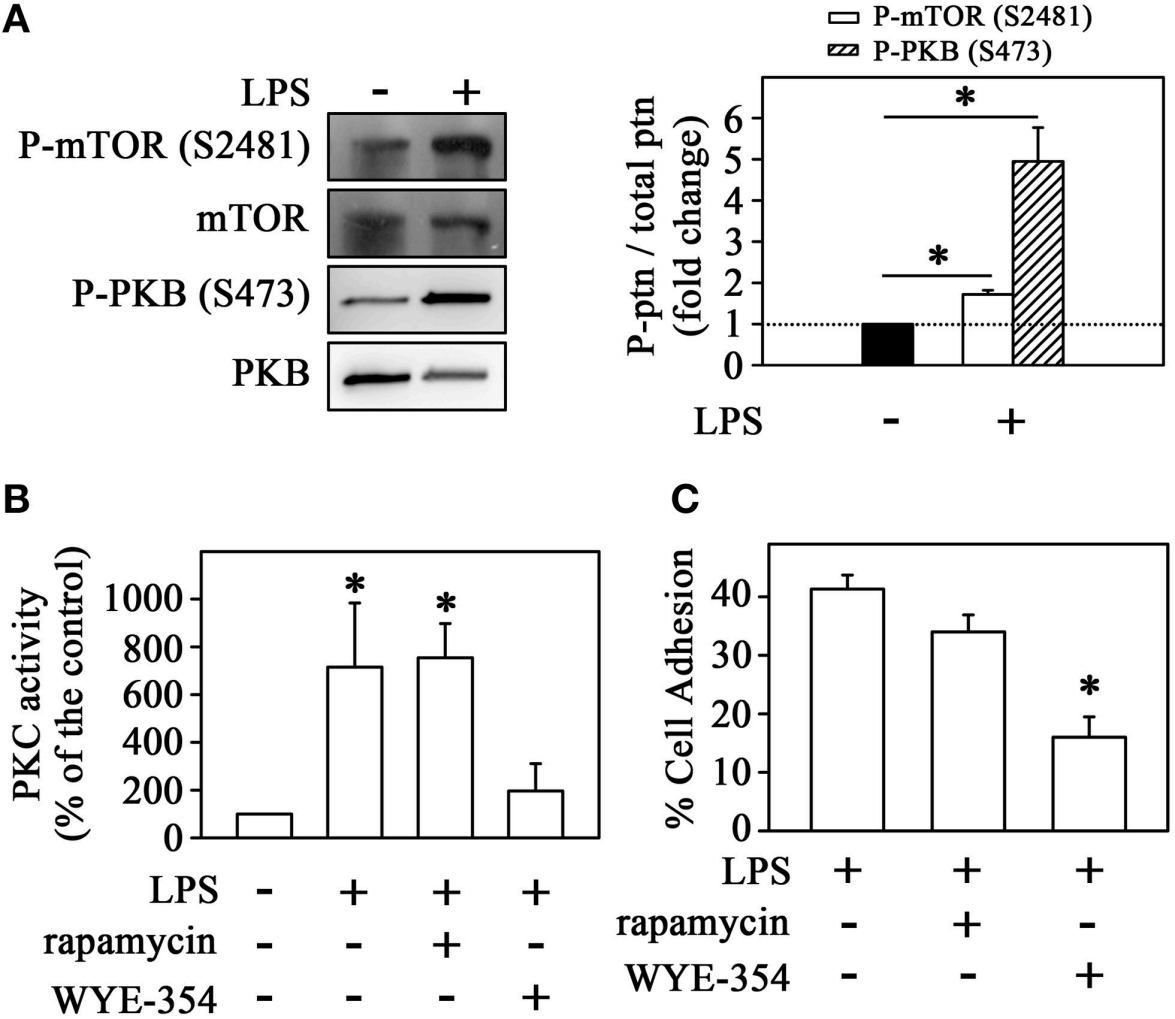
LPS induces the mTORC2/PKC pathway in monocytes. **(A)** The effect of LPS on mTOR and PKB phosphorylation at residues S2481 and S473, respectively (*n* = 3) (left panels). The levels of phosphorylation were determined by the relationship between the optical density of the specific phosphorylated residue and the total fraction. The result is expressed in fold change in relation to an untreated control (bar graph). Images are representative of three independent experiments. The effect of 10^−9^ M rapamycin and 10^−6^ M WYE-354 on **(B)** PKC activity and **(C)** THP-1 cell adhesion induced by LPS (*n* = 3). mTOR, mammalian target of rapamycin; Akt/PKB, protein kinase B. The results are presented as means ± SE. *p < 0.05 vs. unstimulated non-adhered cells (control).

Since mTORC2 can also activate PKC (Hage-Sleiman et al., 2015; Yuan and Guan, 2015), we evaluated the effect of LPS on mTORC2 activity and its involvement in THP-1 cell adhesion. The activation of this complex can be determined by auto-phosphorylation of mTOR at S2481 or phosphorylation of its specific substrate, Akt/PKB, at residue S473 (Laplante and Sabatini, 2012; Peruchetti et al., 2014; Arnaud-Batista et al., 2016). We observed that LPS increased mTORC2 activity after 1 h of incubation measured by both Akt/PKB phosphorylation at residue S473 and mTOR autophosphorylation at residue S2481 (markers of mTORC2 activity) (Figure 2A) as well as PKC activity as shown in Figure 1B. To confirm the involvement of mTORC2 in LPS-induced PKC activity, THP-1 cells were pretreated with either 10^−6^ M WYE-354 (a catalytic inhibitor of both mTORC2 and mTORC1) or 10^−9^ M rapamycin (an mTORC1 inhibitor). We observed that only the pre-incubation with WYE-354 abolished the stimulatory effect of LPS on PKC activity (Figure 2B) and adhesion (Figure 2C). These results suggest that LPS stimulation induces monocyte adhesion through the P13K/mTORC2/PKC signaling pathway.

### The PI3K/mTORC2/PKC pathway triggered by LPS is important to induce CD11a expression

**Figure 3:**
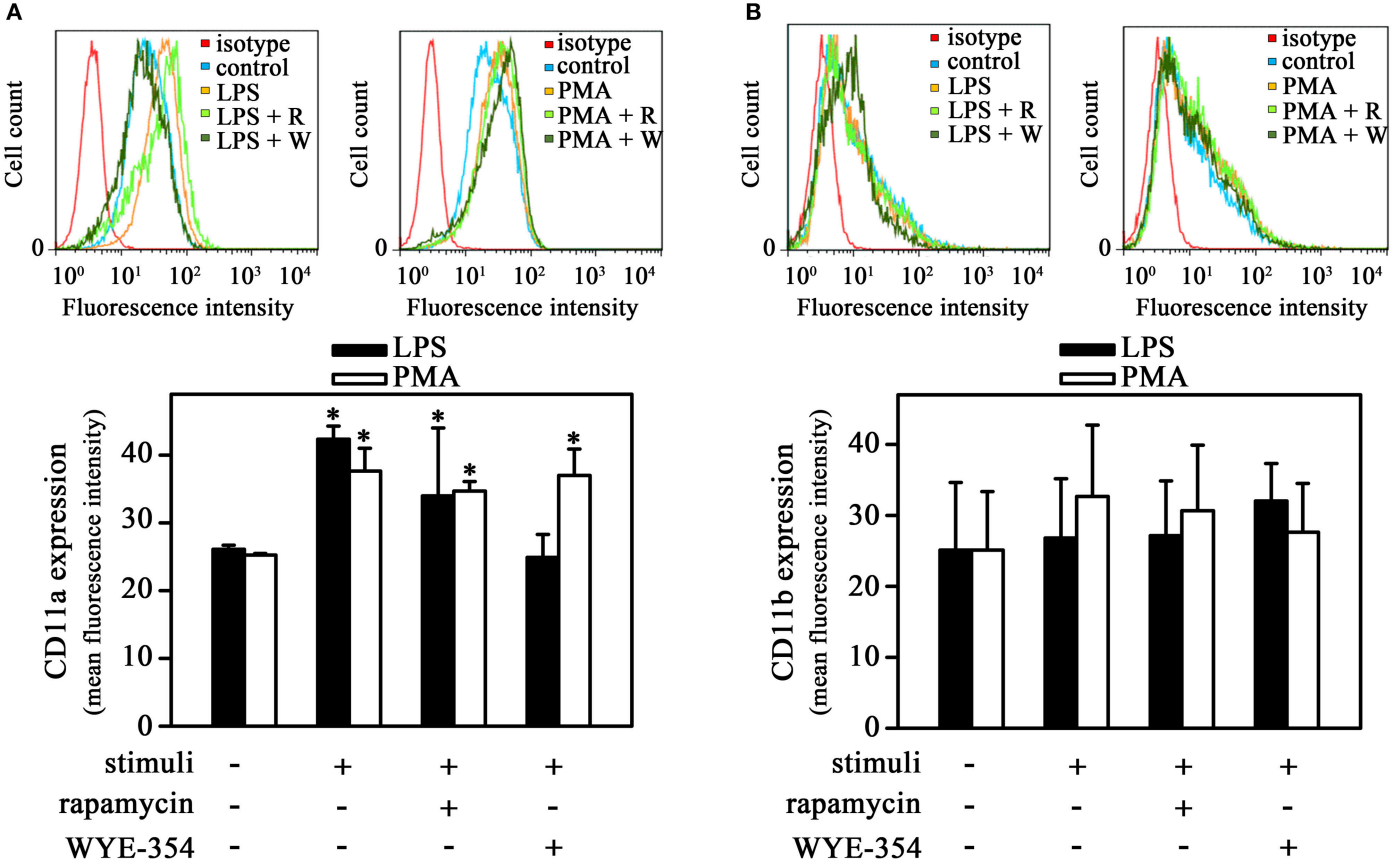
The mTORC2 pathway is involved in CD11a expression induced by LPS. THP-1 cells were pretreated with 10^−9^ M rapamycin or 10^−6^ M WYE-354 for 30 min before incubation with PMA or LPS for 1 h. After treatment, the cells were recovered for reaction with specific antibodies and analyzed by flow cytometry: **(A)** CD11a and **(B)** CD11b expression levels (*n* = 3). The upper panel shows histograms; the bottom panel shows quantification of the mean fluorescence intensity. R, rapamycin; W, WYE-354. The results are presented as means ± SE. *p < 0.05 vs. unstimulated non-adhered cells (control).

β2 integrins such as CD11a and CD11b participate in the process of monocyte transmigration, especially in the adhesion to endothelium (Schenkel et al., 2004). In addition, it has been shown that long-term incubation of LPS and PMA increased both CD11a and CD11b expression in monocytes (Izban et al., 2012; Muñoz-Pacheco et al., 2012; Fogh et al., 2014). Our next step was to verify whether LPS-induced monocyte adhesion involved changes in integrin surface expression in the experimental conditions used. Figure 3A shows that LPS increased CD11a expression in adhered cells after 1 h incubation. The same effect was observed when cells were treated with PMA, which highlights the importance of PKC in this process. Interestingly, LPS-induced CD11a expression was blocked only by WYE-354 but not by rapamycin while PMA-induced CD11a expression was not affected by any inhibitor. On the other hand, there was no modification in CD11b expression in adhered cells (Figure 3B). These results show a role of mTORC2/PKC on CD11a expression during cell adhesion induced by LPS. However, we sought to determine whether the mTOR pathway was involved in cell-cell interaction.

### Monocyte adhesion to ECV-304 cells induced by LPS involves mTOR pathway

**Figure 4:**
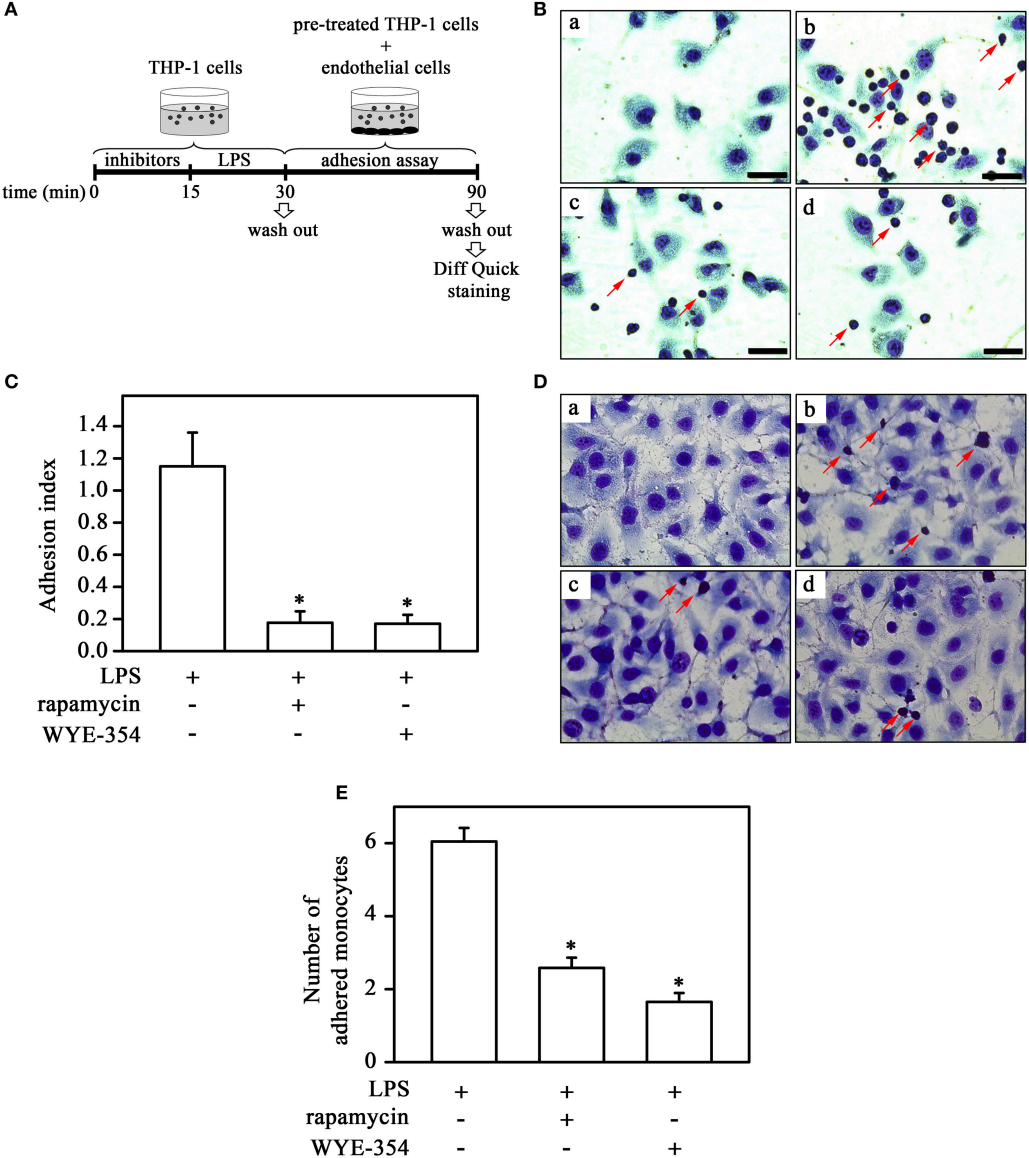
mTORC2 and mTORC1 are involved in monocyte adhesion induced by LPS to ECV cells under static and flow conditions. **(A)** The experimental design is described in detail in the Methods section. Effects of 10^−9^ M rapamycin and 10^−6^ M WYE-354 on THP-1 cell adhesion under static **(B,C)** and flow conditions **(D,E)**. **(B,D)** Images are representative of the experiments. a, control; b, LPS; c, LPS + rapamycin; d, LPS + WYE-354. Red arrows indicate adhered monocytes. Scale bar, 50 μm. **(C,E)** Quantification analyses. The results are presented as means ± SE. *p < 0.05 vs. LPS.

In the next step, we investigated the possible involvement of the mTOR pathway in monocyte-cell adhesion (ECV-304 cells) induced by LPS under static and flow conditions. For this, monocytes were pretreated with mTOR inhibitors as described in the corresponding figure legend before LPS treatment (Figure 4A). In contrast to what was observed for plastic culture plates, under both static and flow conditions, LPS increased THP-1 cell adhesion and pre-incubation with rapamycin or WYE-354 abolished this effect (Figures 4B–E). These results suggest that mTORC1 induces adhesion to ECV cells.

**Figure 5:**
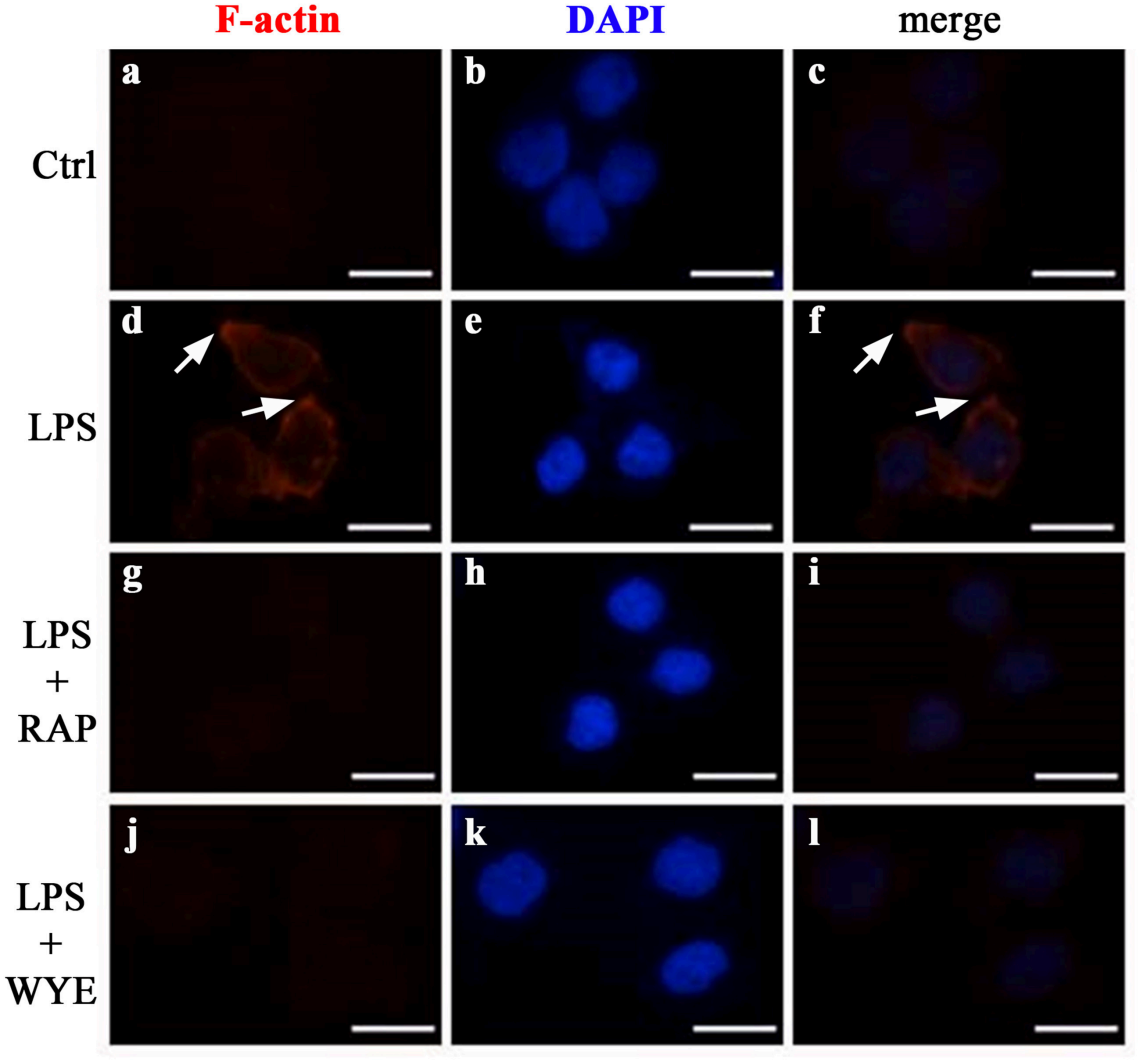
mTORC1 mediates cytoskeleton rearrangement in LPS-induced monocytes. THP-1 cells pretreated with 10^−9^ M rapamycin (RAP) or 10^−6^ M WYE-354 (WYE) before incubation with LPS were allowed to adhere to coverslips coated with 10 μg/mL fibronectin followed by F-actin staining by phalloidin-Alexa Fluor 546 (red color) and DAPI (blue color). **(a–c)** control, **(d–f)** LPS, **(g–i)** LPS + rapamycin, **(j–l)** LPS + WYE-354. Cells were examined by microscopy with 40 × objective. Scale bar, 10 μm (*n* = 3).

Since adhesion involves cytoskeleton rearrangement (Fogh et al., 2014) and mTOR complexes participate in this process (Chen et al., 2015; Sato et al., 2016) we investigated whether mTOR complexes could promote changes in actin polymerization. We observed that non-stimulated THP-1 cells did not exhibit filamentous actin labeling. LPS induced an intense cortical redistribution of actin filaments, as revealed by fluorescent phalloidin staining (Figure 5). In addition, pre-incubation with both rapamycin and WYE-354 completely abolished this effect, indicating the involvement of mTORC1 on cytoskeleton rearrangement induced by LPS.

### Crosstalk between PKC and MEK/ERK/mTORC1 pathways is involved in LPS-induced monocyte adhesion

**Figure 6:**
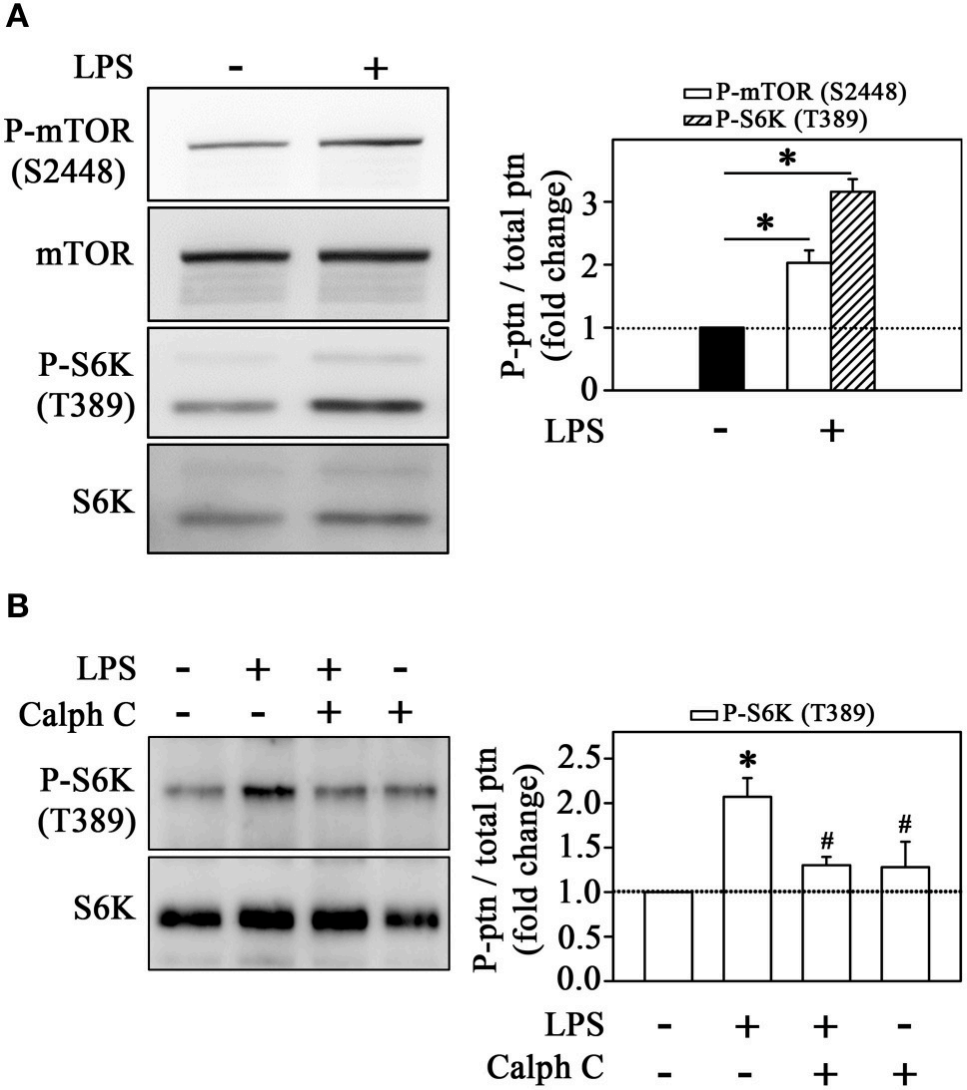
LPS induces the mTORC1/S6K pathway in monocytes. The effects of **(A)** LPS on mTOR and S6K phosphorylation at residues S2448 and T389, respectively (*n* = 3). **(B)** The effect of 10^−6^ M calphostin C on LPS-induced S6K phosphorylation (*n* = 3). The levels of phosphorylation were determined by the relationship between the optical density of specific phosphorylated residues and total fractions. The result is expressed in fold change in relation to an untreated control (bar graph). mTOR, mammalian target of rapamycin; S6K, S6 protein kinase; Akt/PKB, protein kinase B; Calph C, calphostin C. Images are representative of three independent experiments. The results are presented as means ± SE. *p < 0.05 vs. unstimulated non-adhered cells (control). #*p* < 0.05 vs. LPS.

Because our observations showing the involvement of mTORC1 on cytoskeleton rearrangement and monocyte adhesion in the cell-cell adhesion model, we sought to understand the mechanisms underlying LPS-induced mTORC1 activation. Figure 6 shows that LPS increased mTOR phosphorylation in Ser-2448 residue (an mTORC1 activation marker) and S6K phosphorylation at Thr-389 residue, a specific target of mTORC1 (Figure 6A). Because LPS activates PKC, as shown in Figure 1, and it has been already shown that PKC can activate the mTORC1 pathway (Moore et al., 2014), we verified whether the activation of mTORC1 induced by LPS is independent or dependent on PKC activity. Figure 6B demonstrates that PKC mediates the stimulatory effect of LPS on mTORC1 because calphostin C abolished the LPS-induced increase in S6K phosphorylation.

**Figure 7:**
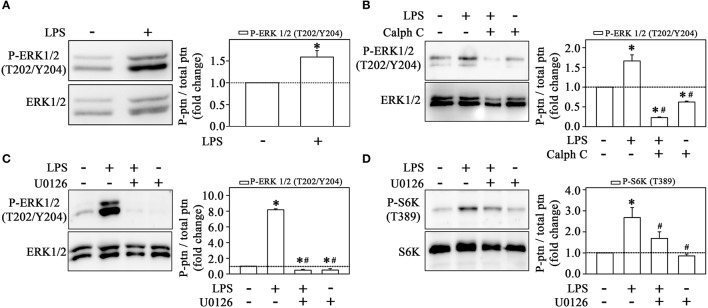
Activation of the mTORC1/S6K pathway induced by LPS is mediated by ERK activation. LPS induces ERK phosphorylation in THP-1 cells (*n* = 3) **(A)**. The effect of 10^−6^ M calphostin C **(B)** or U0126 **(C)** on LPS-induced ERK phosphorylation (*n* = 3). U0126 prevented the effect of LPS on S6K phosphorylation (*n* = 3) **(D)** The levels of phosphorylation were determined by the relationship between the optical density of specific phosphorylated residues and total fractions (left panels). The result is expressed in fold change in relation to an untreated control (bar graph). S6K, S6 protein kinase. Images are representative of three independent experiments. The results are presented as means ± SE. *p < 0.05 vs. unstimulated non-adhered cells (control). #*p* < 0.05 vs. LPS.

Our group and others have shown that ERK activates mTORC1 even in the absence of mTORC2 activation (Winter et al., 2010; Peruchetti et al., 2014). So, to evaluate whether LPS induces the ERK pathway, we determined ERK phosphorylation at residues Thr-202 and Tyr-204. Clearly, strong staining for phospho-ERK was observed after treatment with LPS (Figure 7A). The pretreatment with calphostin C prevented ERK activation, confirming the involvement of PKC in this process (Figure 7B). Moreover, LPS-induced ERK activation was abolished by 10^−6^ M U0126, a MEK inhibitor, which also reduced S6K phosphorylation induced by LPS (Figures 7C,D). These results corroborate the idea that LPS induces MEK/ERK/mTORC1 in a PKC-dependent manner.

**Figure 8:**
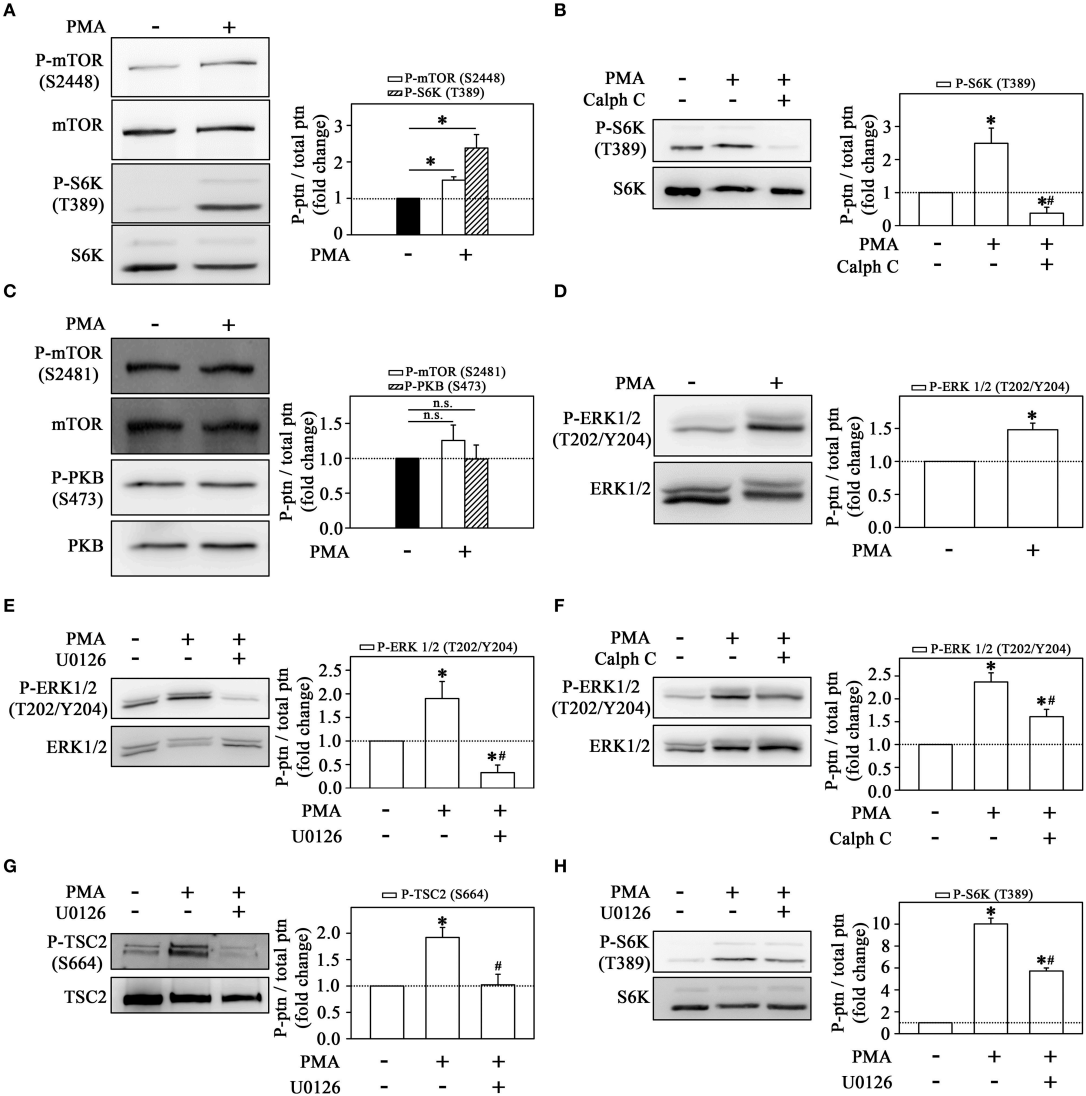
Signaling cascades triggered by the direct activation of PKC on THP-1 monocytes **(A)** PMA induces mTOR and S6K phosphorylation at residues S2448 and T389, respectively (*n* = 3). **(B)** The effect of 10^−6^ M calphostin C on S6K phosphorylation. **(C)** PMA did not change the phosphorylation levels of mTOR on S2481 residue and consequently of Akt/PKB on S473 residue. **(D)** The effect of PMA on ERK phosphorylation. U0126 **(E)** or calphostin C **(F)** prevented the stimulatory effect of PMA on ERK phosphorylation. PMA-induced TSC2 **(G)** and S6K phosphorylation **(H)** is prevented by U0126. The levels of phosphorylation were determined by the relationship between the optical density of specific phosphorylated residues and total fractions. The result is expressed in fold change in relation to an untreated control (bar graph). ERK, extracellular signal-regulated protein kinase; TSC2, tuberous sclerosis complex 2; S6K, S6 protein kinase; Calph C, calphostin C. Images are representative of three independent experiments (left panels). The results are presented as means ± SE. *p < 0.05 vs. unstimulated non-adhered cells (control). #*p* < 0.05 vs. PMA.

One way to demonstrate that the MEK/ERK/mTORC1 pathway is triggered upon PKC activation is to use PMA as a direct PKC activator. With this pharmacological tool, we can delineate the mechanism triggered by LPS, bypassing early signaling events and focusing specifically on downstream events after PKC activation. First, we demonstrated that PMA induced a strong mTORC1 activation as observed in the increase of S6K phosphorylation (Figure 8A). The stimulatory effect of PMA on S6K phosphorylation was completely abolished by calphostin C (Figure 8B). On the other hand, PMA did not change mTORC2 or PKB phosphorylation (Figure 8C). These results led us to propose that PMA-induced mTORC1 activation occurs in a PKC-dependent but mTORC2-independent way.

Also, we observed that PMA increased ERK phosphorylation after 1 h incubation (Figure 8D). This effect of PMA on ERK phosphorylation was reduced by U0126 and calphostin C, suggesting that PMA-induced PKC activates the MEK/ERK pathway (Figures 8E,F).

Activation of ERK leads to inhibition of TSC2 through phosphorylation at residue Ser-664 which, in turn, leads to activation of the mTORC1/S6K pathway (Winter et al., 2010; Peruchetti et al., 2014). As observed in Figure 8G, PMA increased TSC2 phosphorylation, and this effect was completely abolished by pre-incubation with U0126. In addition, the pre-incubation with U0126 also attenuated the stimulatory effect of PMA on S6K phosphorylation (Figure 8H).

**Figure 9:**
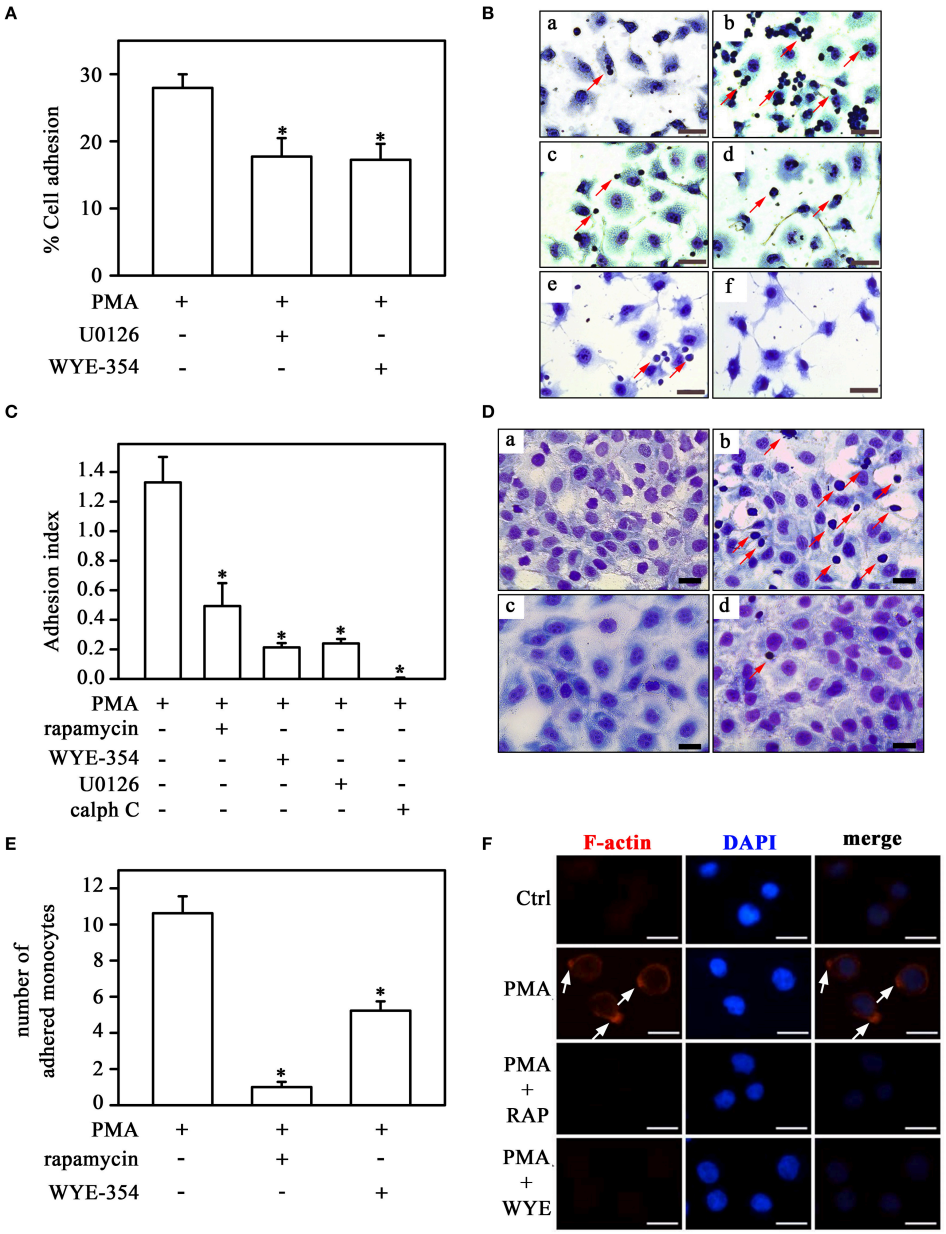
Direct activation of PKC on monocyte adhesion involves the mTORC1/S6K pathway. **(A)** The effect of U0126 and WYE-354 on PMA-induced THP-1 cell adhesion (*n* = 3). **(B,C)** Effects of rapamycin, WYE-354, U0126, and calphostin C on PMA-induced THP-1 cell adhesion under static conditions. **(D,E)** Effects of rapamycin and WYE-354 on PMA-induced THP-1 cell adhesion under flow conditions. **(B,D)** Images are representative of the experiments. a, control; b, LPS; c, LPS+rapamycin; d, LPS+WYE-354; e, LPS+U0126; f, LPS+calph C. Red arrows indicate adhered monocytes. Scale bar, 50 μm. **(C,E)** Quantification analyses. **(F)** Effect of rapamycin and WYE-354 on PMA-induced THP-1 cells on cytoskeleton rearrangement as described in Figure 5. (a–c) control, (d–f) PMA treatment, (g–i) PMA and rapamycin treatment, (j–l) PMA and WYE-354 treatment. Cells were examined by microscopy with 40 × objective. Scale bar, 10 μm (*n* = 3). Calph C, calphostin C; RAP, rapamycin; WYE, WYE-354. The results are presented as means ± SE. *p < 0.05 vs. PMA.

The impact of the PKC/MEK/ERK/mTORC1 pathway on the adhesion mechanism was assessed in Figure 9. PMA-induced THP-1 cell adhesion to plastic culture plates was inhibited by U0126 and WYE-354 (Figure 9A). In addition, calphostin C, WYE-354, rapamycin and U0126 reduced adhesion to ECV-304 cells triggered by PMA (Figures 9B,C). Furthermore, mTOR inhibitors also blocked the effect of PMA on adhesion under flow conditions (Figures 9D,E) and on fiber actin polarization (Figure 9F).

## Discussion

mTORC1 and mTORC2 are involved in several cell functions and regulatory mechanisms (Laplante and Sabatini, 2012; Shimobayashi and Hall, 2014). Here, we demonstrated the role of PKC as a central molecular link in the activation of such complexes in response to LPS stimuli. Our results demonstrated that LPS triggered a wide-spectrum intracellular signaling cascade that leads to cytoskeleton rearrangement and upregulation of integrin expression culminating in monocyte adhesion.

The participation of PKC and PI3K in LPS responses have been reported (Williams and Ridley, 2000; Jones and Okeoma, 2013; Xie et al., 2014; Kim et al., 2015; Filippi, 2016; Dwyer et al., 2017). However, the interdependence between these signaling proteins in response to LPS is poorly described. Previously, our group demonstrated that PKC phosphorylation and activity were dependent on previous activation of PI3K in proximal tubule cells (Peruchetti et al., 2014). Accordingly, in our first group of results, we observed that LPS-induced PKC activity was abolished by PI3K inhibitor. In the same condition, we observed a reduction in monocyte adhesion at levels similar to the condition where PKC is inhibited. These results allowed us to suggest that the activation of PKC induced by LPS occurs in a PI3K-dependent manner and this process is associated with THP-1 monocyte adhesion.

Classically, PKB is firmly recognized as an effector of PI3K in cells. Activation of PKB is achieved when phosphorylation of S473 in the hydrophobic motif occurs (Manning and Toker, 2017). The primary kinase involved in this process is mTORC2 (Manning and Toker, 2017). Therefore, detection of phosphorylated PKB in S473 is a way of determining activation of mTORC2. Under the experimental conditions used, we observed an increase in PKB phosphorylation, suggesting LPS-induced mTORC2 activity. Moreover, the inhibition of mTORC2 activity, but not of mTORC1, blocked the effect of LPS on PKC activity and adhesion on plastic culture plates. The direct activation of PKC mediated by mTORC2 has been proposed in other systems (Su and Jacinto, 2011; Hage-Sleiman et al., 2015; Yuan and Guan, 2015). Thus, our findings highlight the activation of the PI3K/mTORC2/PKC pathway in response to LPS stimuli. What is the relevance of this signaling mechanism in terms of monocyte adhesion?

Cell adhesion is a key step in cell migration and has been explored extensively in different research fields (Imhof and Aurrand-Lions, 2004). The integrin family of proteins function as cell surface receptors and promote interaction between cells and extracellular matrix proteins or mediate leukocyte-endothelial cell interactions commonly through the recognition of ICAM-1, the ligand expressed on the surface of endothelial cells (Giagulli et al., 2004; Souza et al., 2012). Data from the literature have shown that PKC activity is directly associated with cell adhesion, although this process may or may not be dependent on changes in CD11a expression levels (Izban et al., 2012; Muñoz-Pacheco et al., 2012; Fogh et al., 2014). Our data showed that LPS as well as PMA, a PKC activator, increased CD11a expression in THP-1 cells. Moreover, the inhibition of mTORC2, but not of mTORC1, abolished the effect of LPS on CD11a expression, suggesting the involvement of the mTORC2/PKC pathway in integrin expression and, consequently, adhesion triggered by LPS. In agreement, in an elegant study on cancer cell migration, Chen et al. (2015) reported that mTORC2 regulates cell adhesion through an Akt-independent mechanism, very similar to what we describe here.

Although ECV-304 cells are not recognized as endothelial cells because they lack VCAM, E-selectin and PECAM-1 (Lidington et al., 1999), they are widely used as a model to study adhesion mechanisms dependent on ICAM-1 (Böger et al., 2000; Souza et al., 2012; Hien et al., 2014). As we observed different results in adhesion assays performed in culture plate and ECV-304 cells, it is possible to postulate that these differences are due to the absence of receptor-ligand recognition mechanism in culture plate. In the present work, although we have detected activation of mTORC1 in cells adhered to cultured plates, rapamycin (which works as a specific mTORC1 inhibitor under the experimental conditions used) did not influence LPS-induced adhesion in the same experimental set. However, under both static and flow conditions, we observed that not only WYE-354 but also rapamycin completely blocked LPS-induced THP-1 cell interaction with the cell monolayer. Moreover, both inhibitors completely blocked the formation of focal adhesion. These results give us the idea that mTORC1 is clearly involved in LPS-induced monocyte adhesion. However, we cannot rule out the possible involvement of mTORC2 in this process, since WYE-354 inhibits both mTOR complexes and mTORC2 involvement in adhesion was well-characterized by the results obtained on culture plates. Accordingly, Chen et al. (2015) have also shown that both complexes are essential for cell adhesion in breast cancer cells. Whether the activation of mTORC1, and possibly mTORC2, on monocytes, and consequently, cell adhesion is a direct effect of LPS or is dependent on the release of certain soluble factors will require further experiments.

In the present work, we observed that LPS activated the mTORC1/S6K pathway in monocytes. But the molecular mechanism underlying the activation of mTORC1 triggered by LPS is still unknown. Because both PKC and mTORC1 are activated by LPS, it is plausible to imagine a crosstalk between those pathways. Gibbs et al. (2011) showed that SCF-induced HIFα expression, an important molecule involved in cell adaptation under conditions of low oxygen availability, was mediated by activation of the PKCδ/mTORC1 pathway in THP-1 cells. Similarly, we demonstrated that mTORC1 activation was dependent on previous activation of PKC. This signaling cascade underlying mTORC1 activation was further evaluated using PMA, a pharmacological tool to directly activate PKC and mimic the effects of LPS. Interestingly, we observed that PMA also activated the mTORC1/S6K pathway and induced THP-1 cell adhesion. Moreover, calphostin C (a PKC inhibitor) blocked the stimulatory effect of PMA on mTORC1 activity and THP-1 adhesion to ECV cells. In agreement with our findings, Minhajuddin et al. (2009) showed that PKCδ is constitutively associated with mTORC1 and modulates its activity triggered by thrombin in endothelial cells.

At this moment, one question arises: how does PKC activate the mTORC1/S6K pathway triggered by LPS? It has been shown that peptidoglycan-induced CCL2 and CCL4 secretion involves activation of both ERK and mTORC1 in THP-1 cells (Lee et al., 2011). In addition, Kampen et al. (2014), using phospho-proteome (kinome) and immunoblotting analyses, showed that MEK inhibitor reduced the phosphorylation of S6K at residue Thr-424 (a specific target of mTORC1) and residue Thr-229 (T loop) in THP-1 cells. The first is located in the C-terminal region of S6K, which is important to configure an open structure allowing access of mTORC1 to the hydrophobic motif (Magnuson et al., 2012). Based on this evidence, it is plausible to imagine that ERK1/2 may be a link between PKC and mTORC1 activities induced by LPS. Here, we observed that LPS or PMA increased ERK1/2 and mTORC1 activity (assessed by S6K phosphorylation) and this effect was abolished by calphostin C (a PKC inhibitor) and U0126 (a MEK/ERK inhibitor). In addition, the MEK inhibitor also blocked the effect of PMA on monocyte adhesion. These results allowed us to postulate that LPS induces monocyte adhesion through the mTORC2/PKC pathway, which in turn leads to activation of the ERK/mTORC1/S6K pathway. In a previous work, our group showed that higher albumin concentrations induced overactivation of the mTORC1/S6K pathway mediated by ERK/TSC2 activity in proximal tubule cells [20]. Here, we observed that TSC2 also mediates ERK-induced activation of mTORC1 in THP-1 cells.

PKC exists in at least 12 different known isoforms distributed in three distinct classes: conventional (cPKC), novel (nPKC), and atypical (aPKC) (Hage-Sleiman et al., 2015; Fleming and Storz, 2017). To explore the involvement of PKC in the effects of LPS, we used two well-known pharmacological tools: (1) PMA, a phorbol ester that activates both cPKCs and nPKCs by binding in the C1 regulatory domain (mimicking diacylglycerol [DAG]); and (2) calphostin C, which specifically inhibits PKCs by competing with the C1 regulatory domain with PMA or DAG (EMD Millipore, #208725). Analyzing the data of the present work, we observed that PMA mimicked the effects of LPS in several results. In addition, Figure 1B shows that incubation of THP-1 cells with LPS increased a calphostin C-sensitive PKC activity. Together this evidence suggests that LPS-induced monocyte adhesion involves PKC isoforms that belong to the cPKC and/or nPKC classes, but not the aPKC class. Further experiments are necessary to identify which specific PKC isoform is involved in LPS-induced monocyte adhesion.

**Figure 10:**
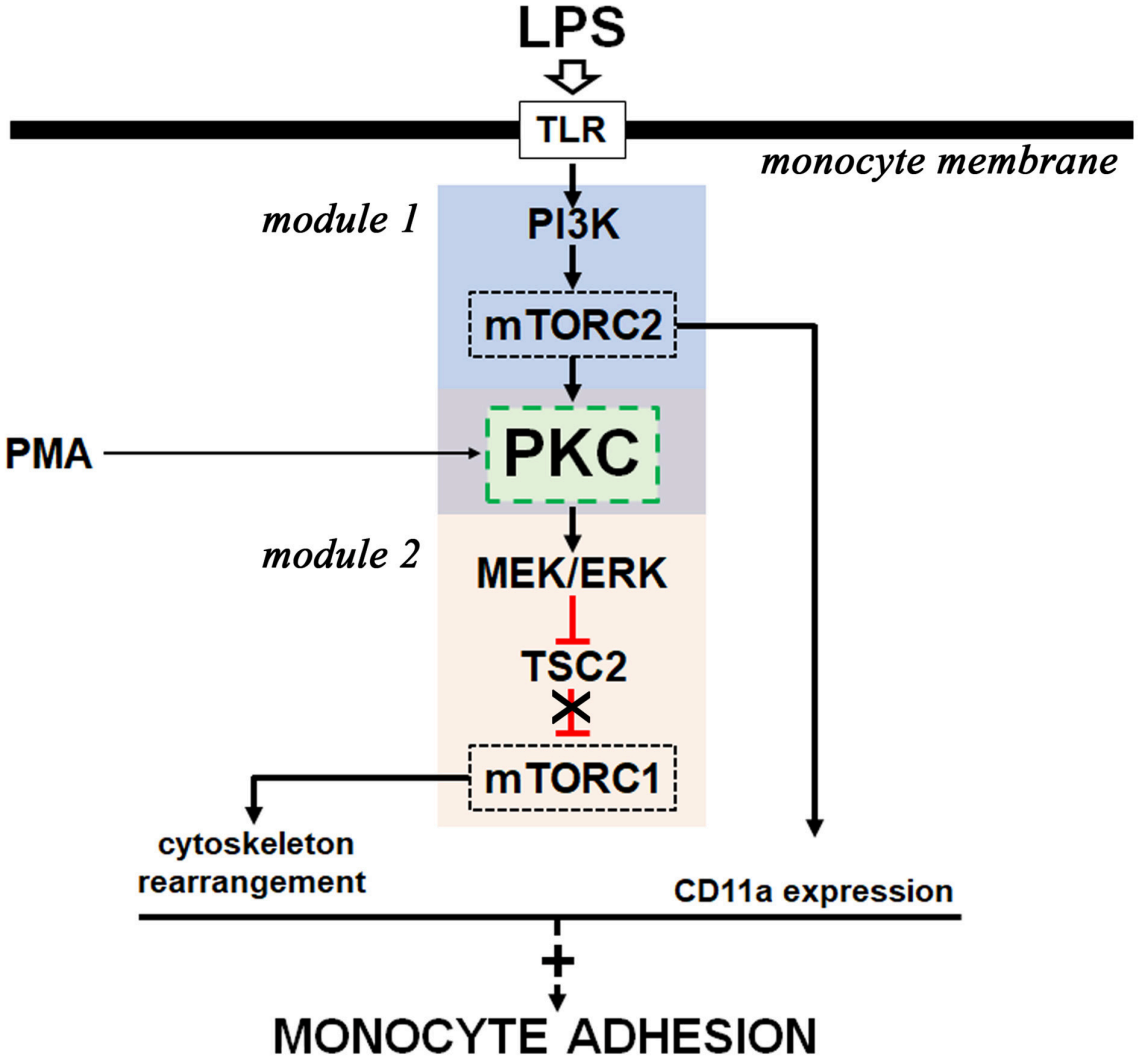
Proposed model for the molecular mechanism involved in LPS-induced monocyte adhesion. LPS activates two signaling modules (module 1 and module 2) with the involvement of PKC activity (green box). Black arrows indicate activation signals and red symbols indicate inhibitory signals. A detailed explanation is given in the text.

Taken together, our results describe an intricate signaling cascade induced by LPS involved in THP-1 cell adhesion (Figure 10). In our system, we have evidence of the activation of two distinct pathways perfectly linked by PKC: one is the PI3K/mTORC2/PKC pathway, which leads to activation of the ERK/TSC2/mTORC1 cascade in response to LPS. The activation of both mTOR complexes is directly involved in THP-1 adhesion by regulating the expression of CD11a and the formation of focal adhesion.

## Conclusions

The results in the present manuscript describe a wide spectrum signaling cascade induced by LPS in THP-1 cells. These results add new understanding of the molecular mechanisms underlying monocyte adhesion, opening new avenues for possible therapeutic intervention of immune-based diseases in which monocyte adhesion has a critical role.

## Author contributions

AP and CC-N gave substantial contributions to the conception and design of the work. All experiments were designed by AP, CC-N, MS, and MH. MR, DP, LS, JS-F carried out experiments and improved protocols. All authors analyzed data. AP, CC-N, DP, MR, and LS wrote the manuscript and all authors have read and revised it critically. All authors agreed and approved the final version of the manuscript to be submitted for publication.

### Conflict of interest statement

The authors declare that the research was conducted in the absence of any commercial or financial relationships that could be construed as a potential conflict of interest.

## Abbreviations

ERK: extracellular-signal-regulated kinase
LPS: lipopolysaccharide
MEK: mitogen-activated protein kinase kinase
mTOR: mammalian target of rapamycin
mTORC1: mammalian target of rapamycin complex 1
mTORC2: mammalian target of rapamycin complex 2
PKB: protein kinase B
PKC: protein kinase C
PMA: phorbol 12-myristate 13-acetate
S6K: S6 kinase
THP-1: human acute monocytic leukemia cell line.
