# Supplementary figures and images for: LPS Induces mTORC1 and mTORC2 Activation During Monocyte Adhesion
Source: Front Mol Biosci. 2018 Jul 18;5:67. doi: 10.3389/fmolb.2018.00067 (PMC6058081; doi:10.3389/fmolb.2018.00067)

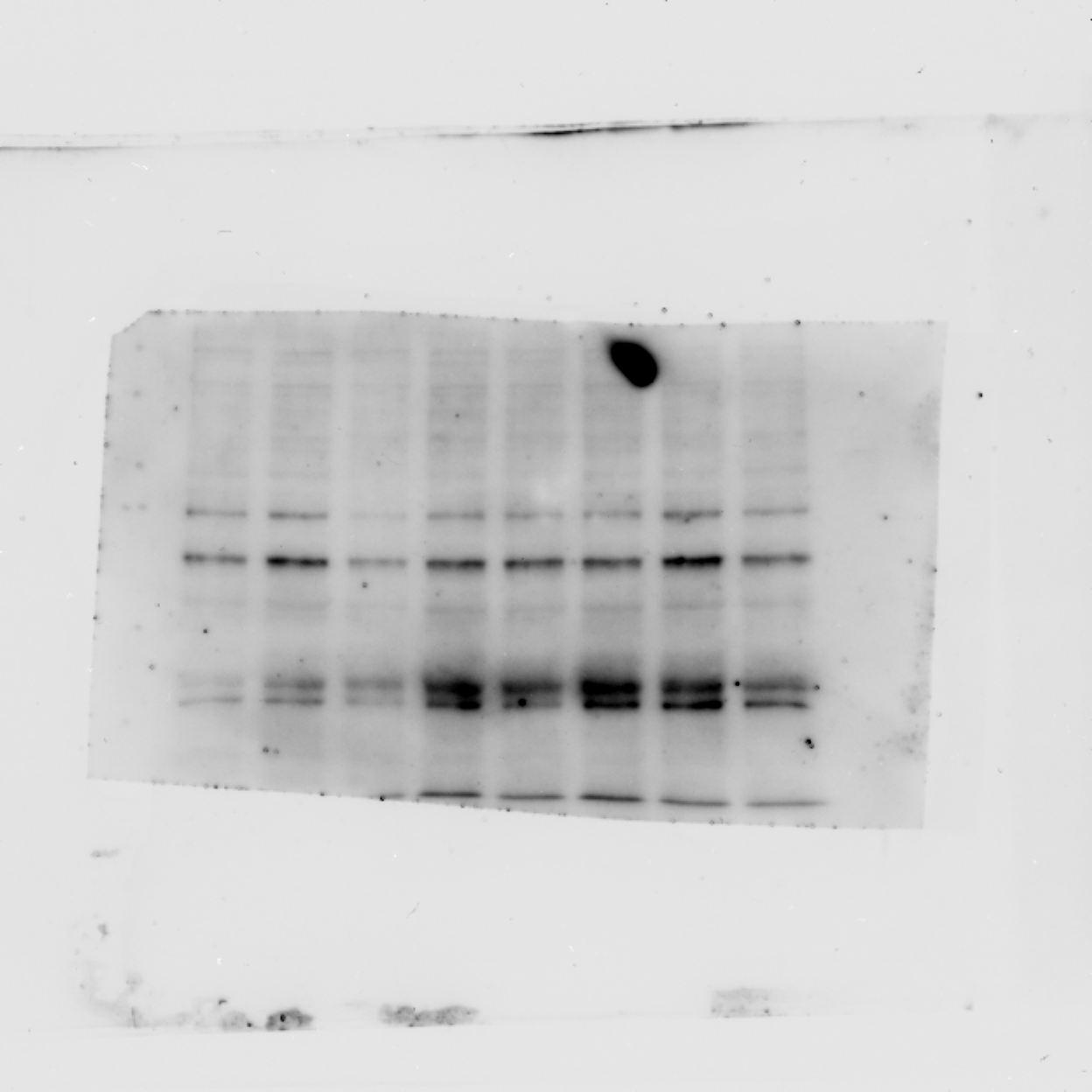

Supplement: Supplementary file 1 [file Data_Sheet_1.ZIP › 327021_images_1/Fig 6B P-S6K (T389).tif]

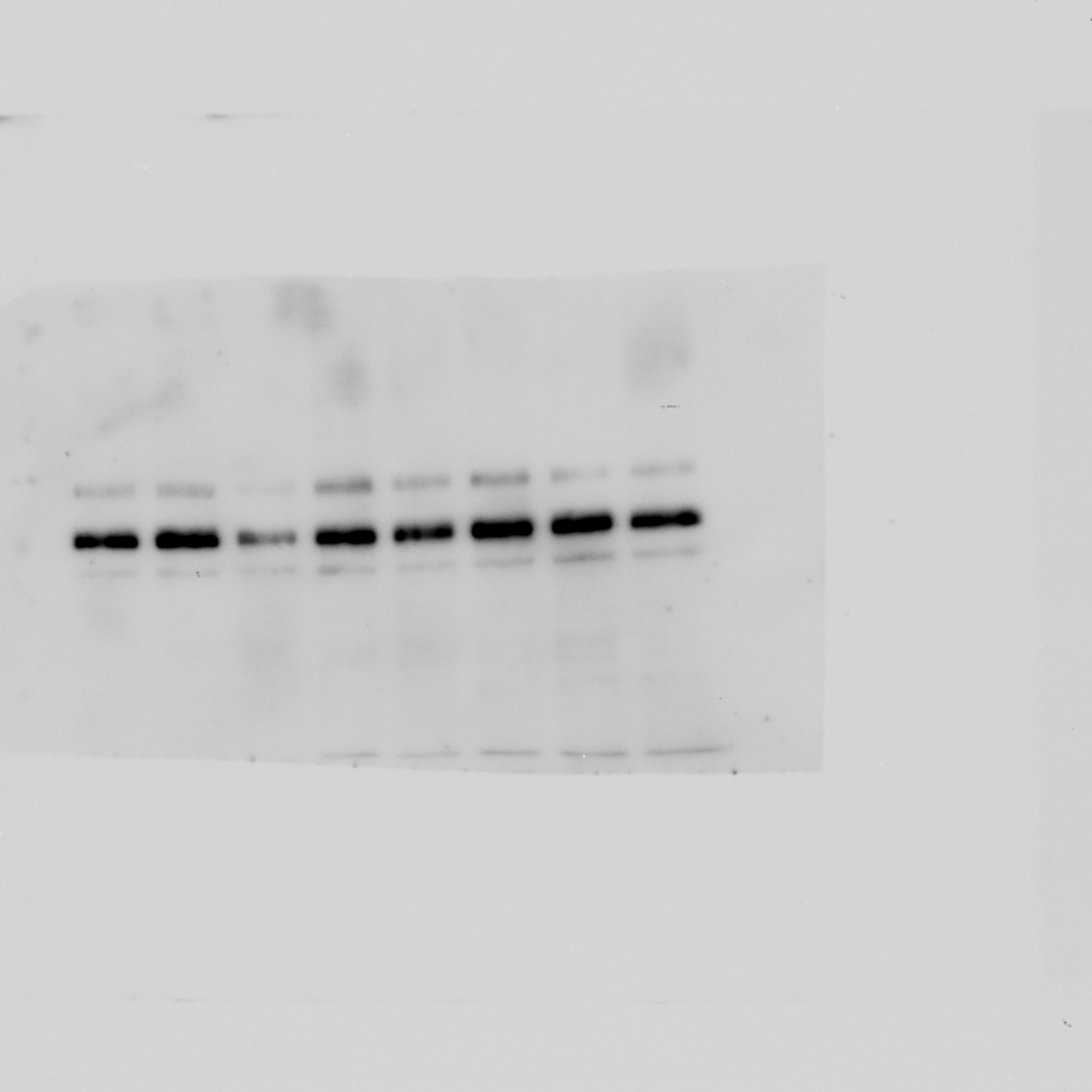

Supplement: Supplementary file 1 [file Data_Sheet_1.ZIP › 327021_images_1/Fig 6B S6K.tif]

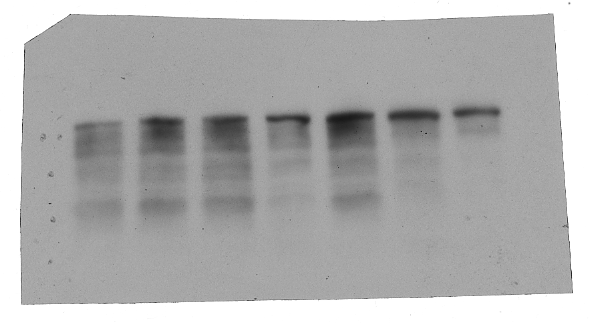

Supplement: Supplementary file 1 [file Data_Sheet_1.ZIP › 327021_images_1/Figure 2A mTOR.tif]

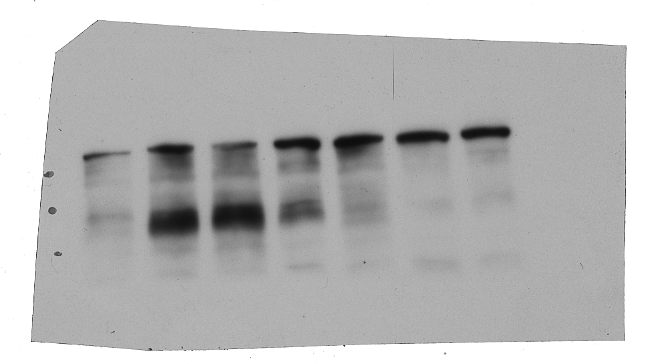

Supplement: Supplementary file 1 [file Data_Sheet_1.ZIP › 327021_images_1/Figure 2A P-mTOR (S2481).tif]

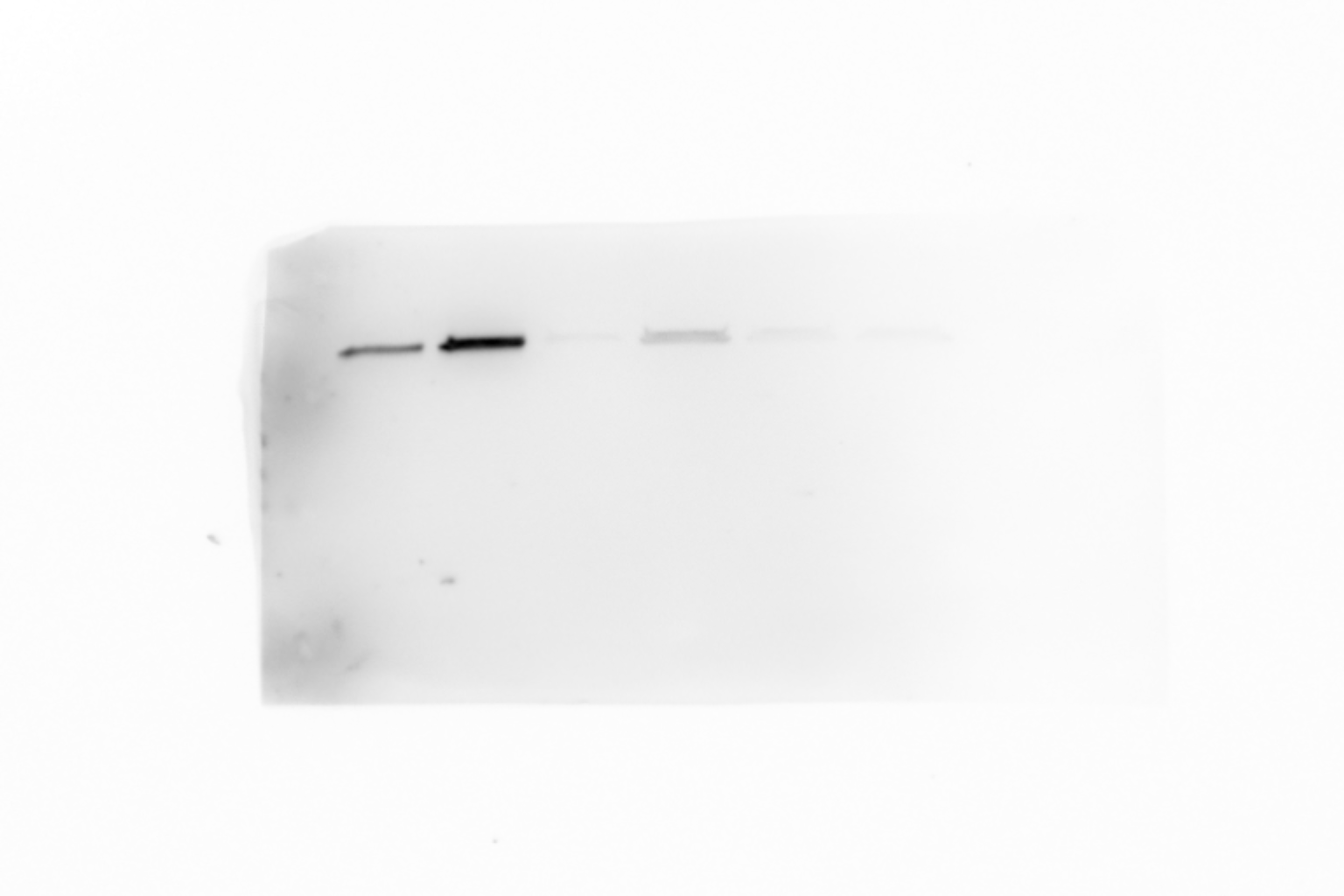

Supplement: Supplementary file 1 [file Data_Sheet_1.ZIP › 327021_images_1/Figure 2A P-PKB (S473).tif]

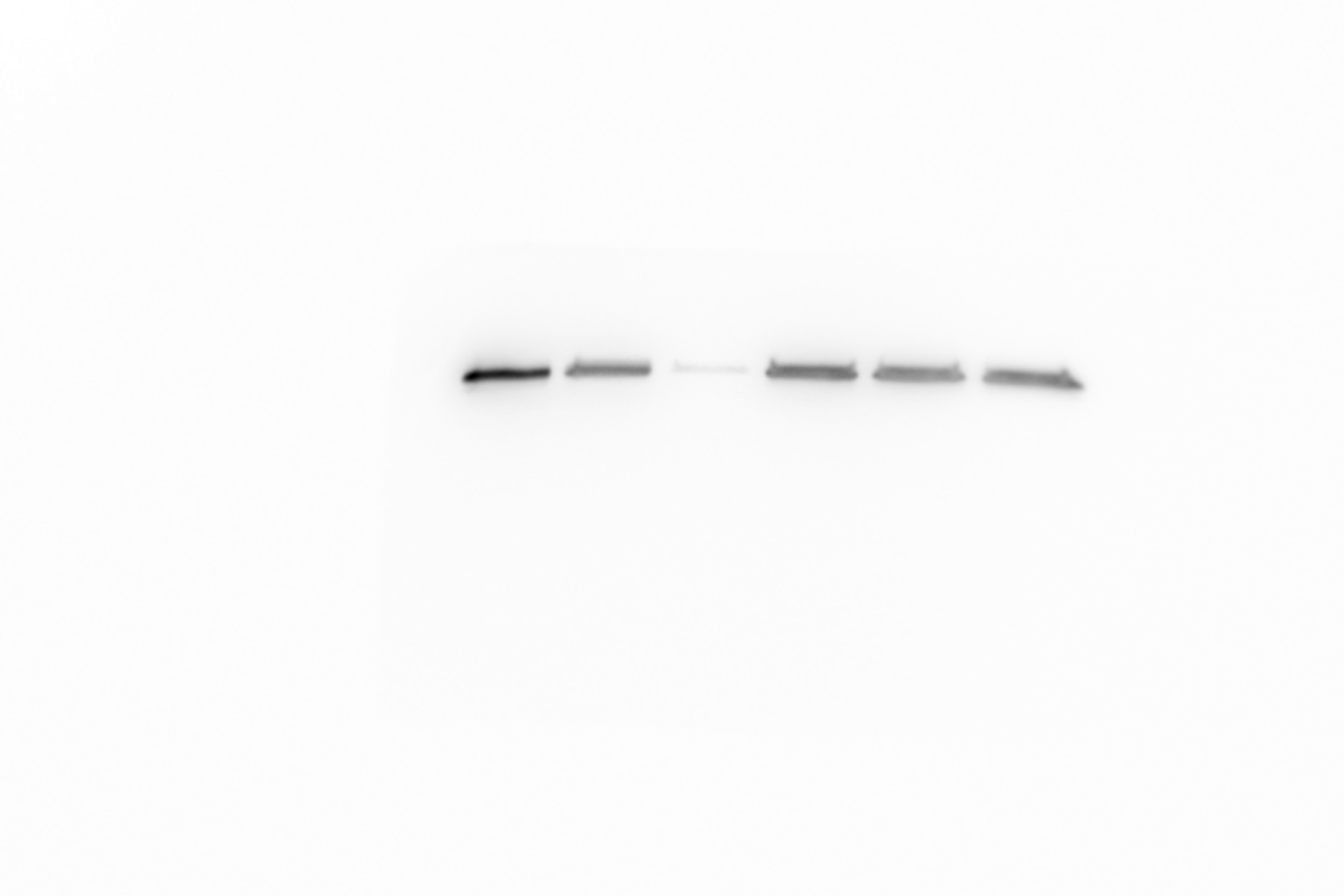

Supplement: Supplementary file 1 [file Data_Sheet_1.ZIP › 327021_images_1/Figure 2A PKB.tif]

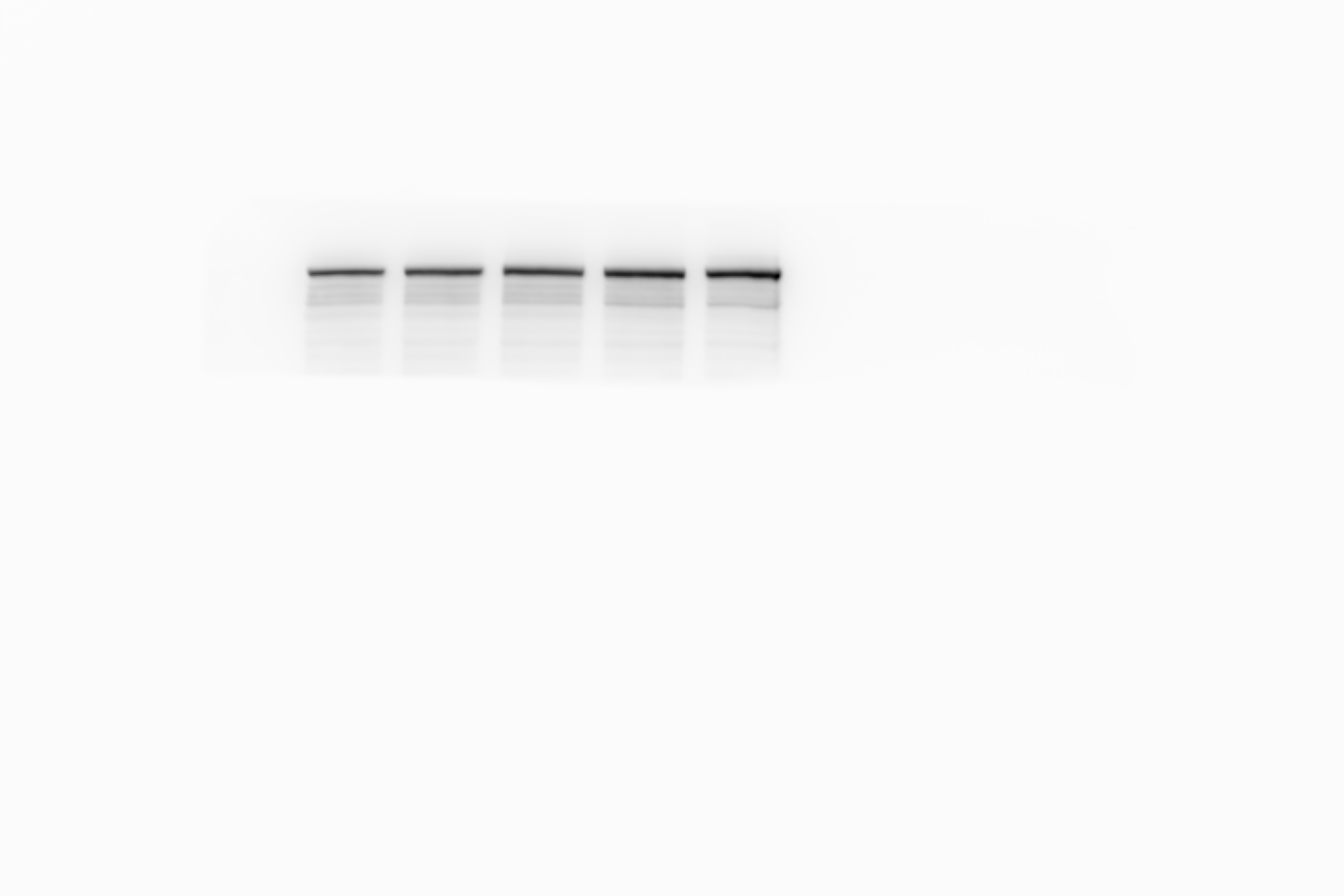

Supplement: Supplementary file 1 [file Data_Sheet_1.ZIP › 327021_images_1/Figure 6A mTOR.tif]

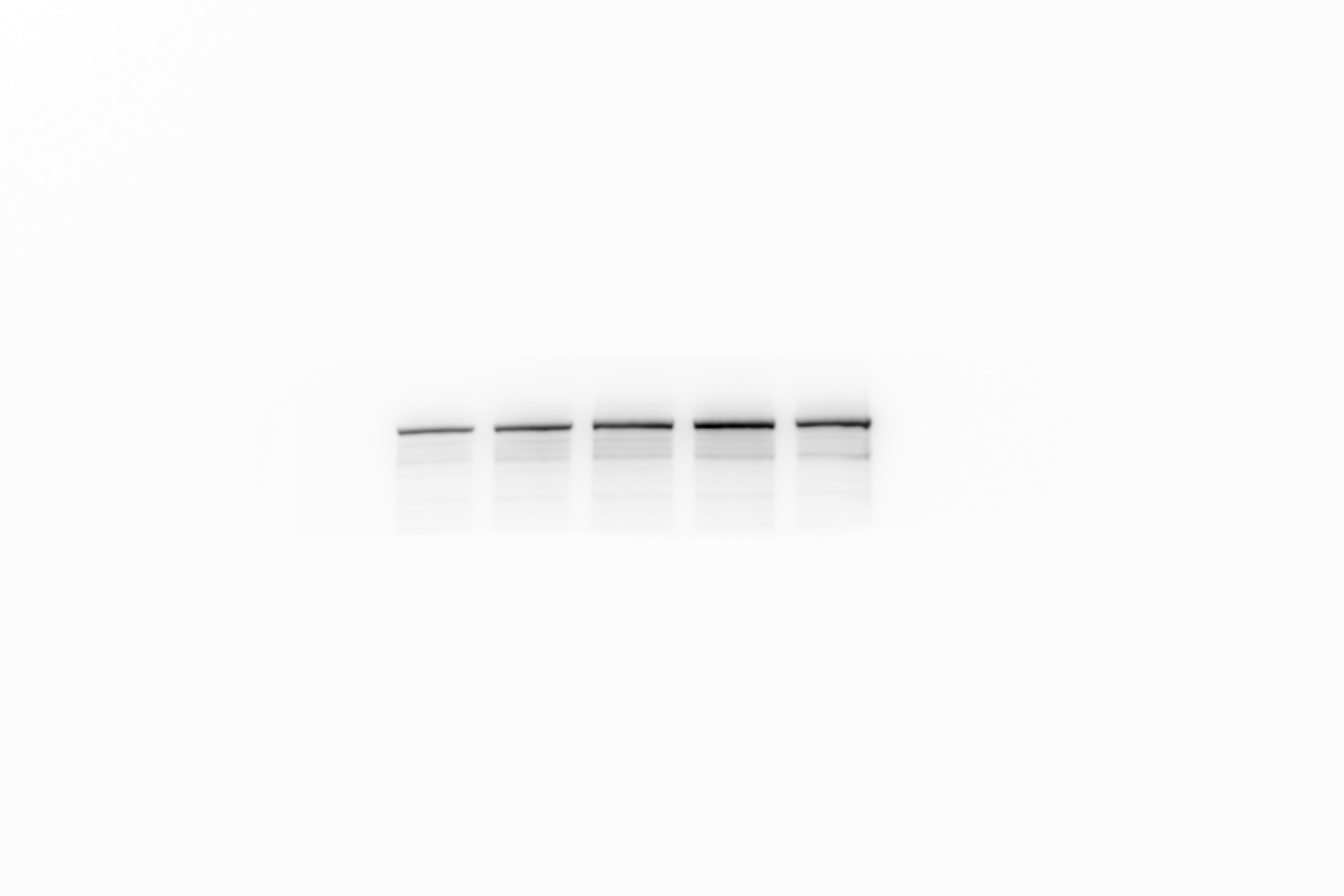

Supplement: Supplementary file 1 [file Data_Sheet_1.ZIP › 327021_images_1/Figure 6A P-mTOR (S2448).tif]

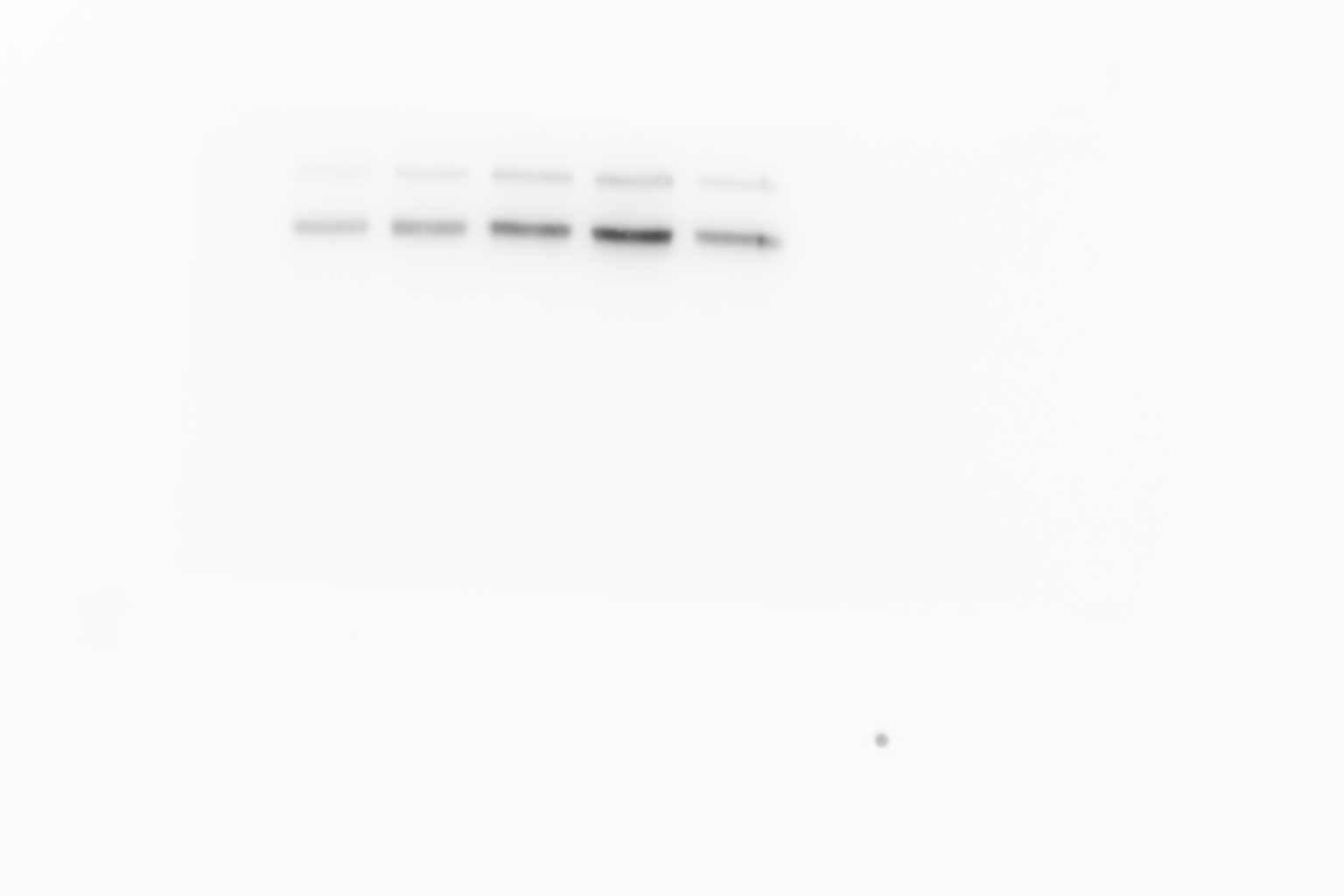

Supplement: Supplementary file 1 [file Data_Sheet_1.ZIP › 327021_images_1/Figure 6A P-S6K (T389).tif]

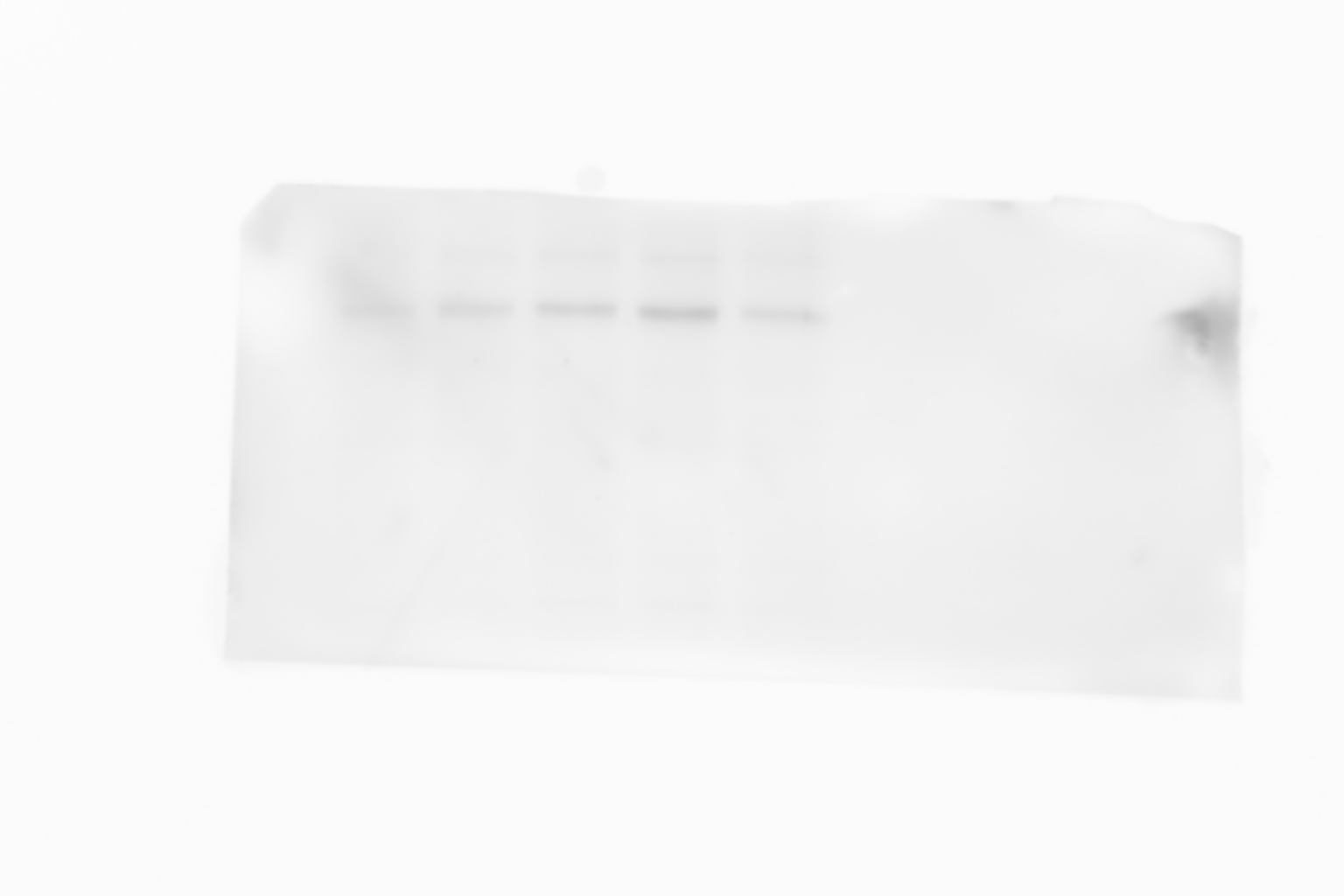

Supplement: Supplementary file 1 [file Data_Sheet_1.ZIP › 327021_images_1/Figure 6A S6K.tif]

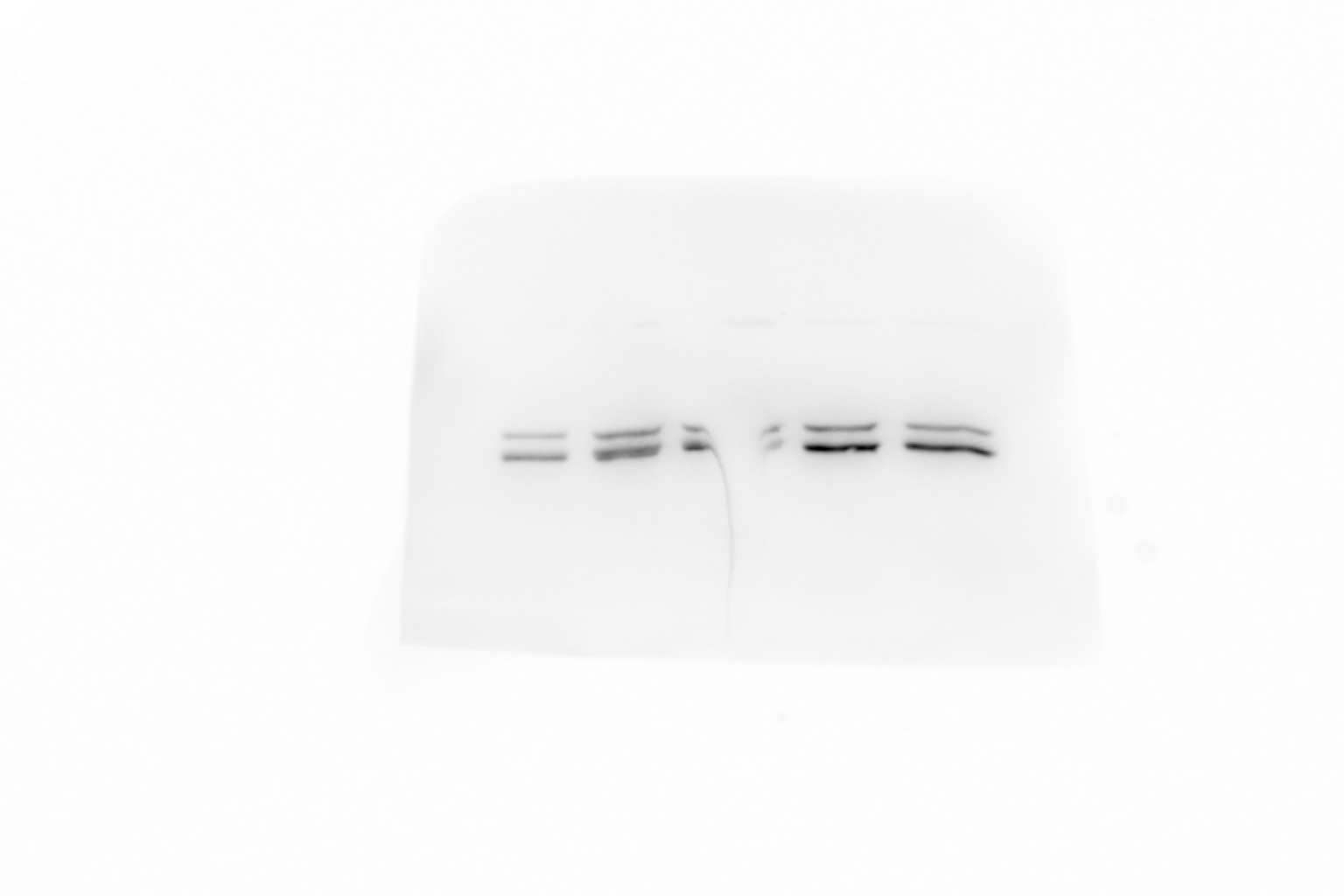

Supplement: Supplementary file 2 [file Data_Sheet_2.ZIP › 327021_images_2/Figure 7A ERK1_2.tif]

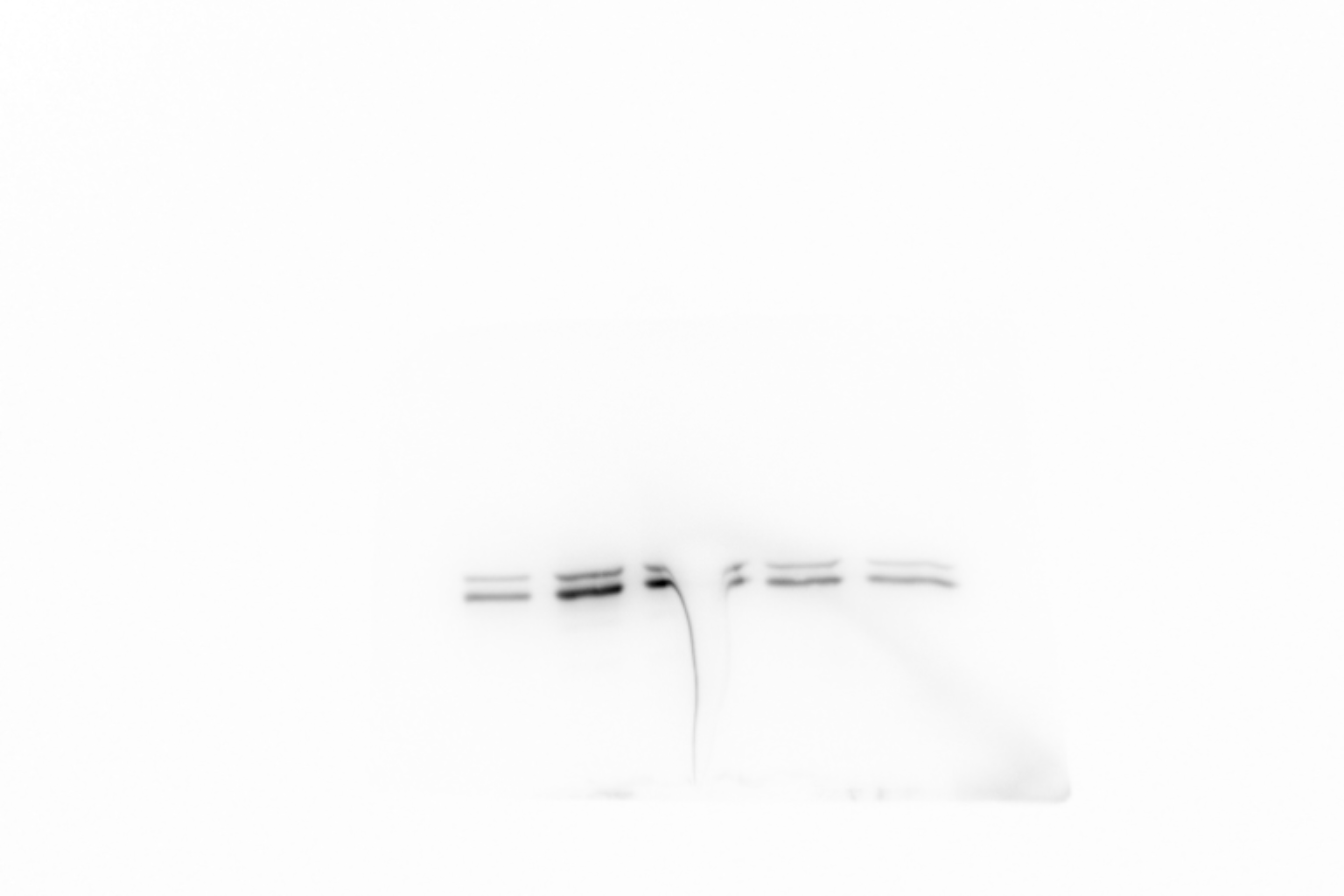

Supplement: Supplementary file 2 [file Data_Sheet_2.ZIP › 327021_images_2/Figure 7A P-ERK1_2 (T202_Y204).tif]

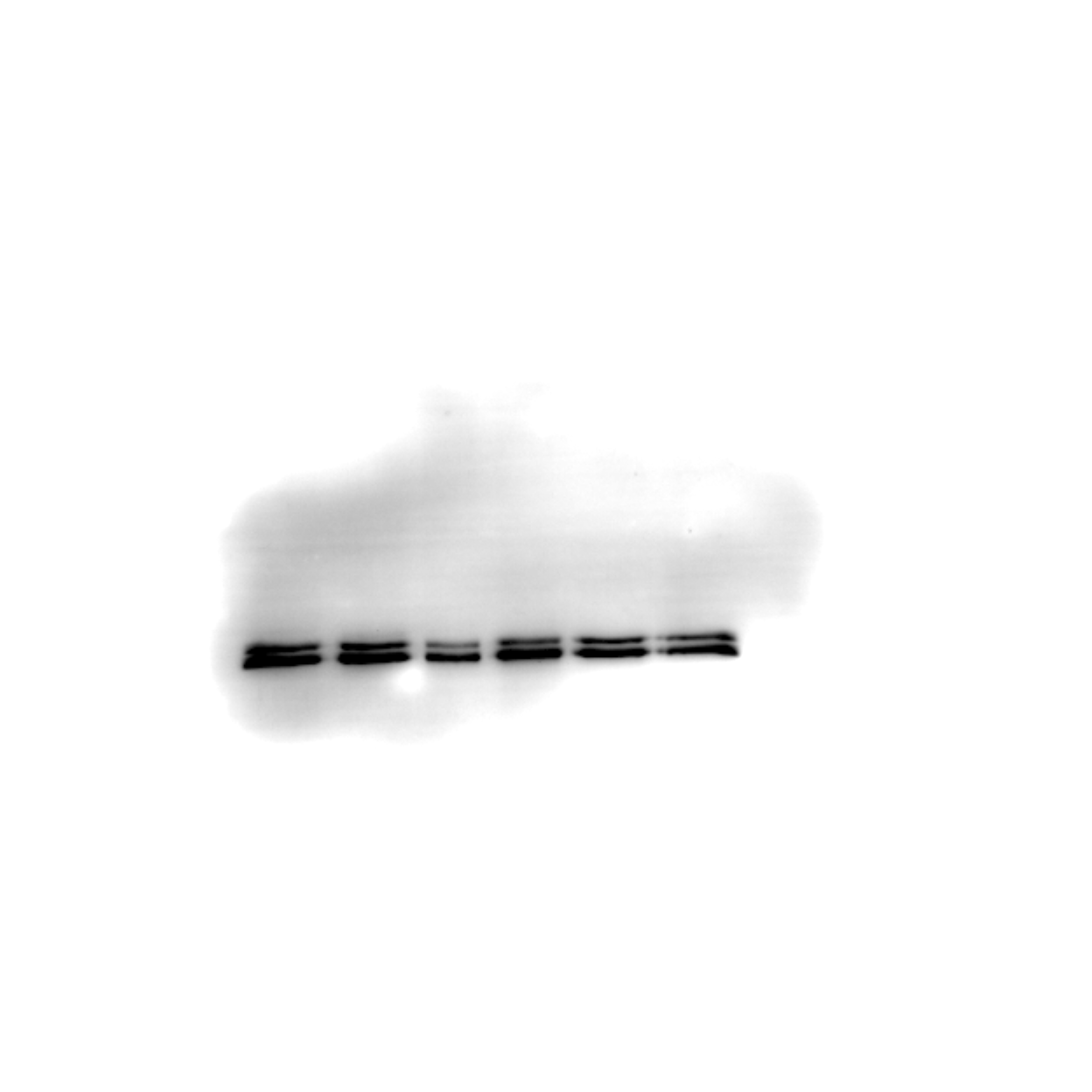

Supplement: Supplementary file 2 [file Data_Sheet_2.ZIP › 327021_images_2/Figure 7B ERK.tif]

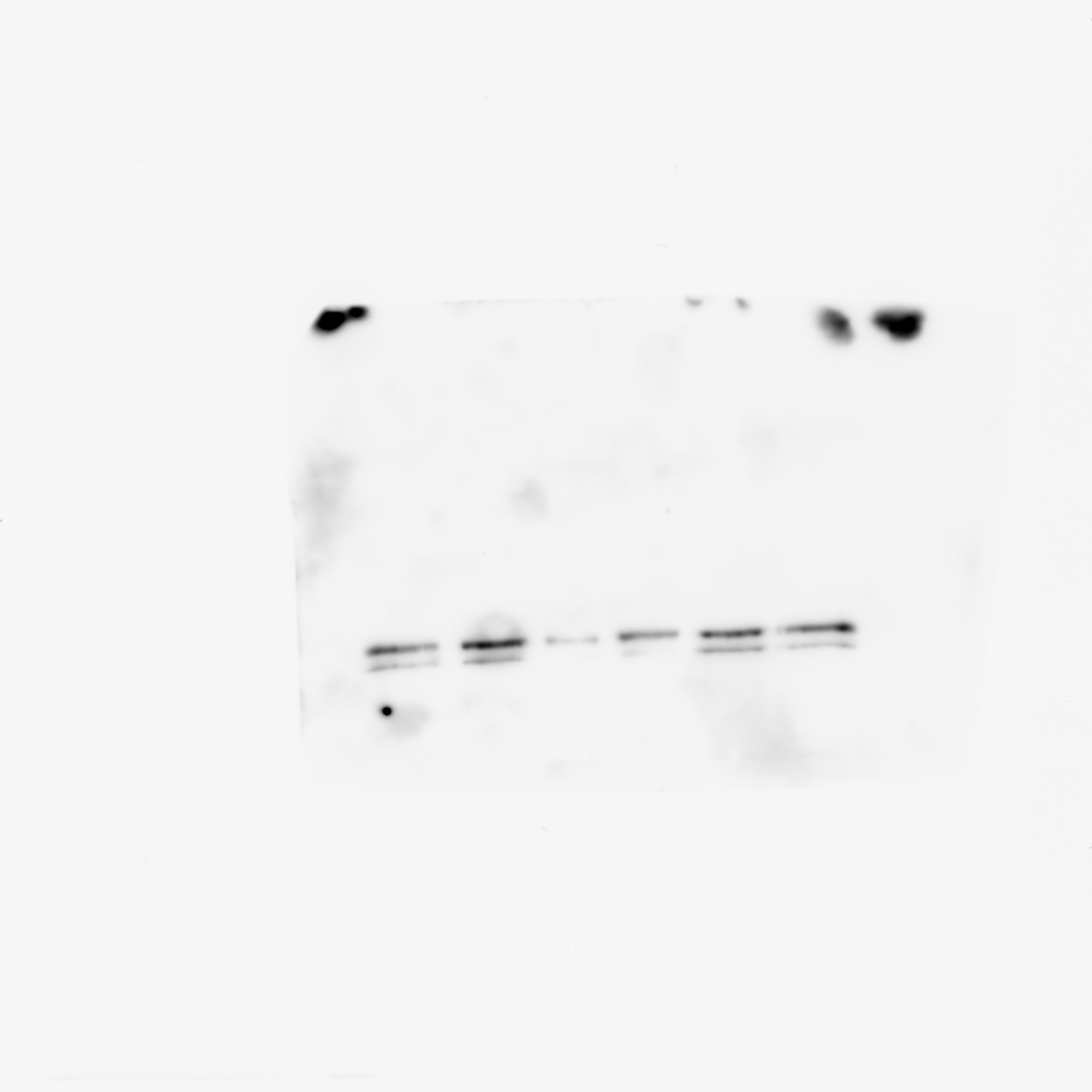

Supplement: Supplementary file 2 [file Data_Sheet_2.ZIP › 327021_images_2/Figure 7B P-ERK.tif]

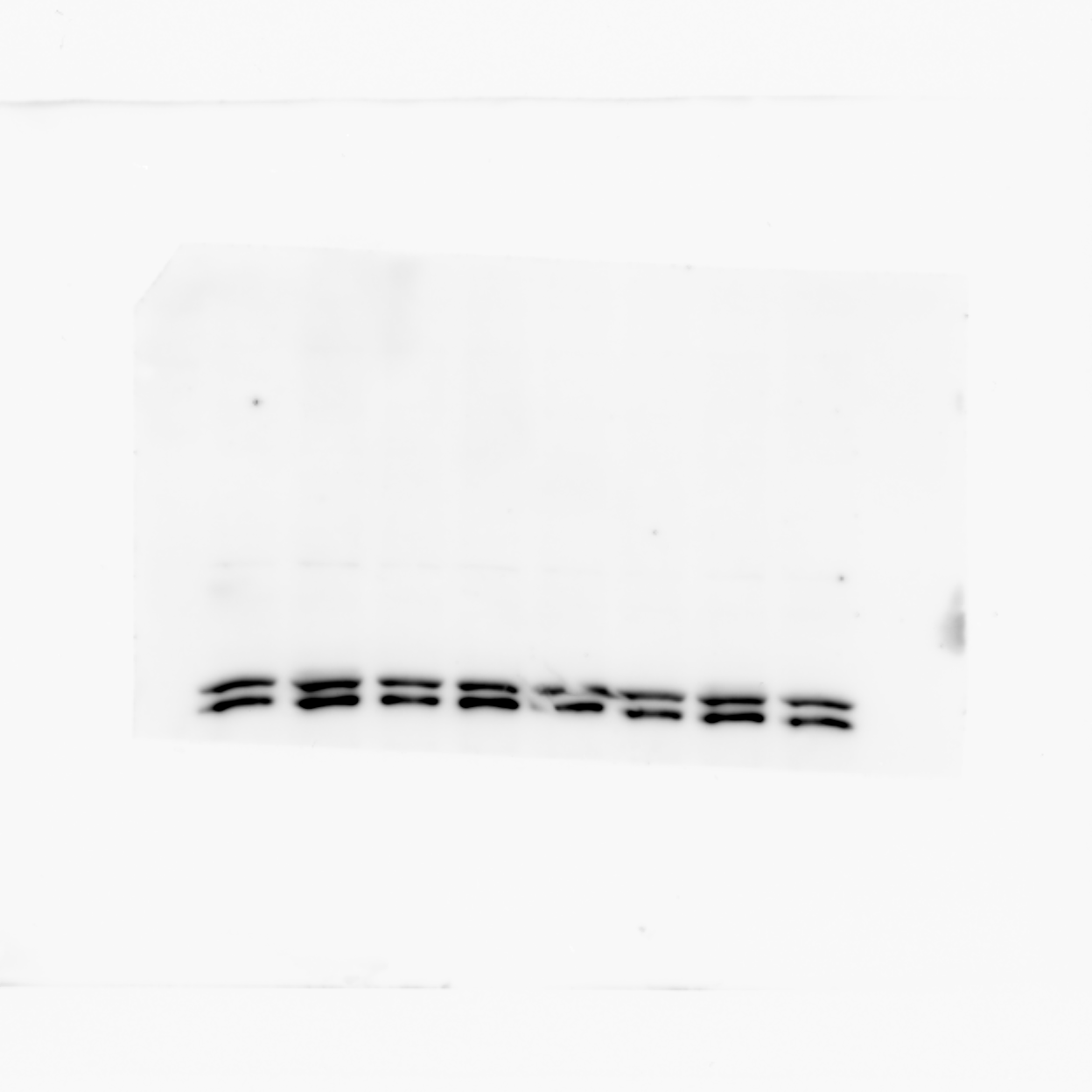

Supplement: Supplementary file 2 [file Data_Sheet_2.ZIP › 327021_images_2/Figure 7C ERK.tif]

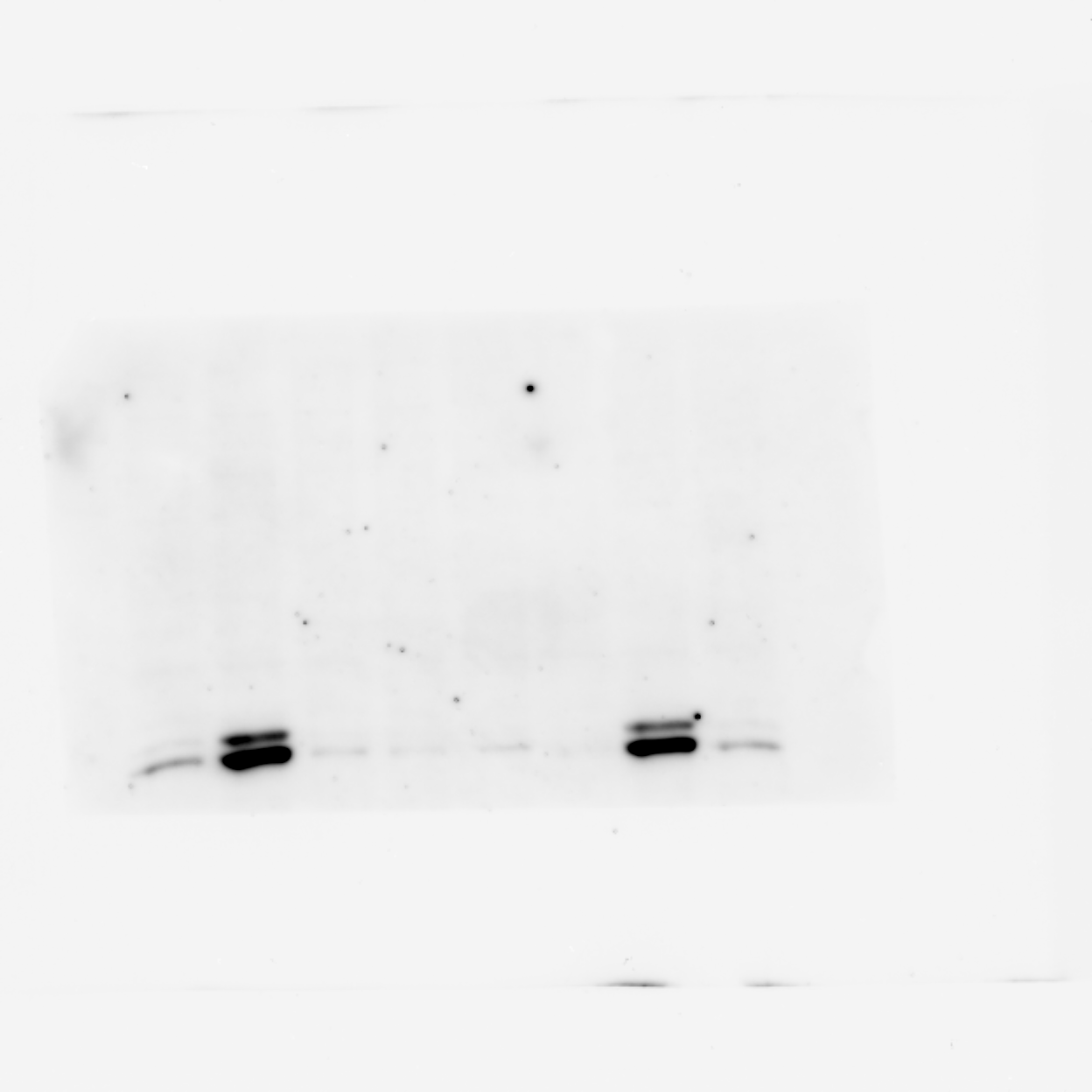

Supplement: Supplementary file 2 [file Data_Sheet_2.ZIP › 327021_images_2/Figure 7C P-ERK.tif]

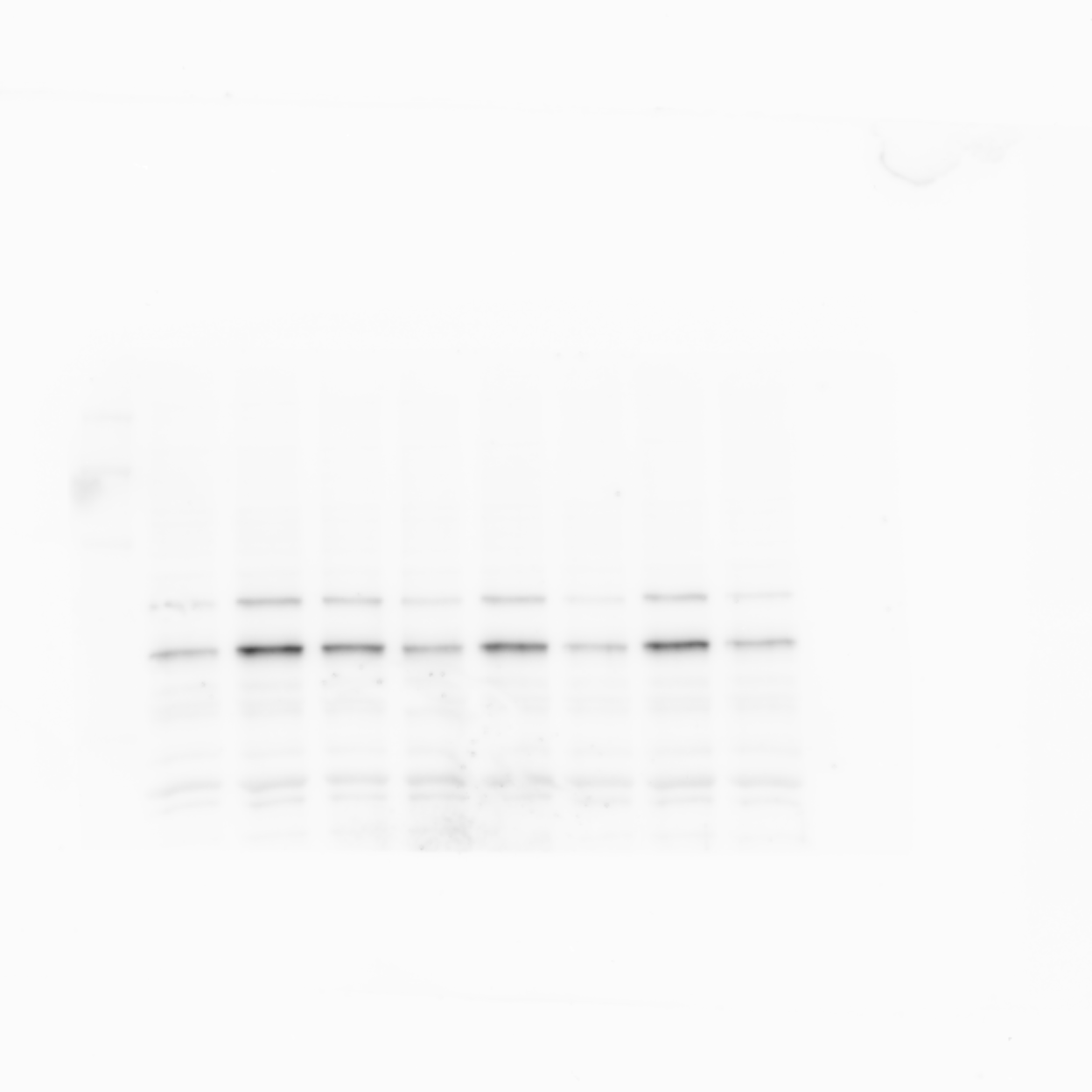

Supplement: Supplementary file 2 [file Data_Sheet_2.ZIP › 327021_images_2/Figure 7D P-S6K.tif]

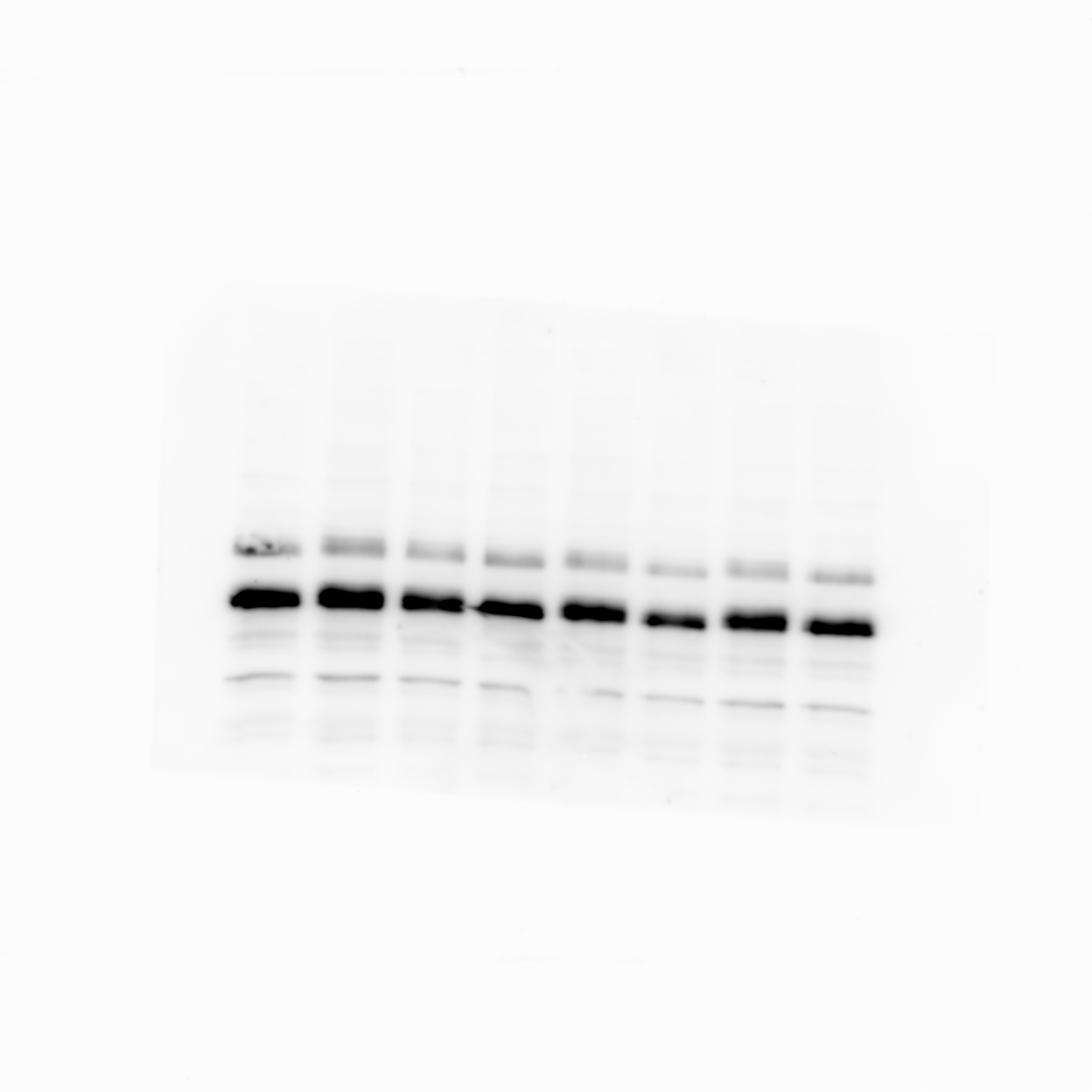

Supplement: Supplementary file 2 [file Data_Sheet_2.ZIP › 327021_images_2/Figure 7D S6K.tif]

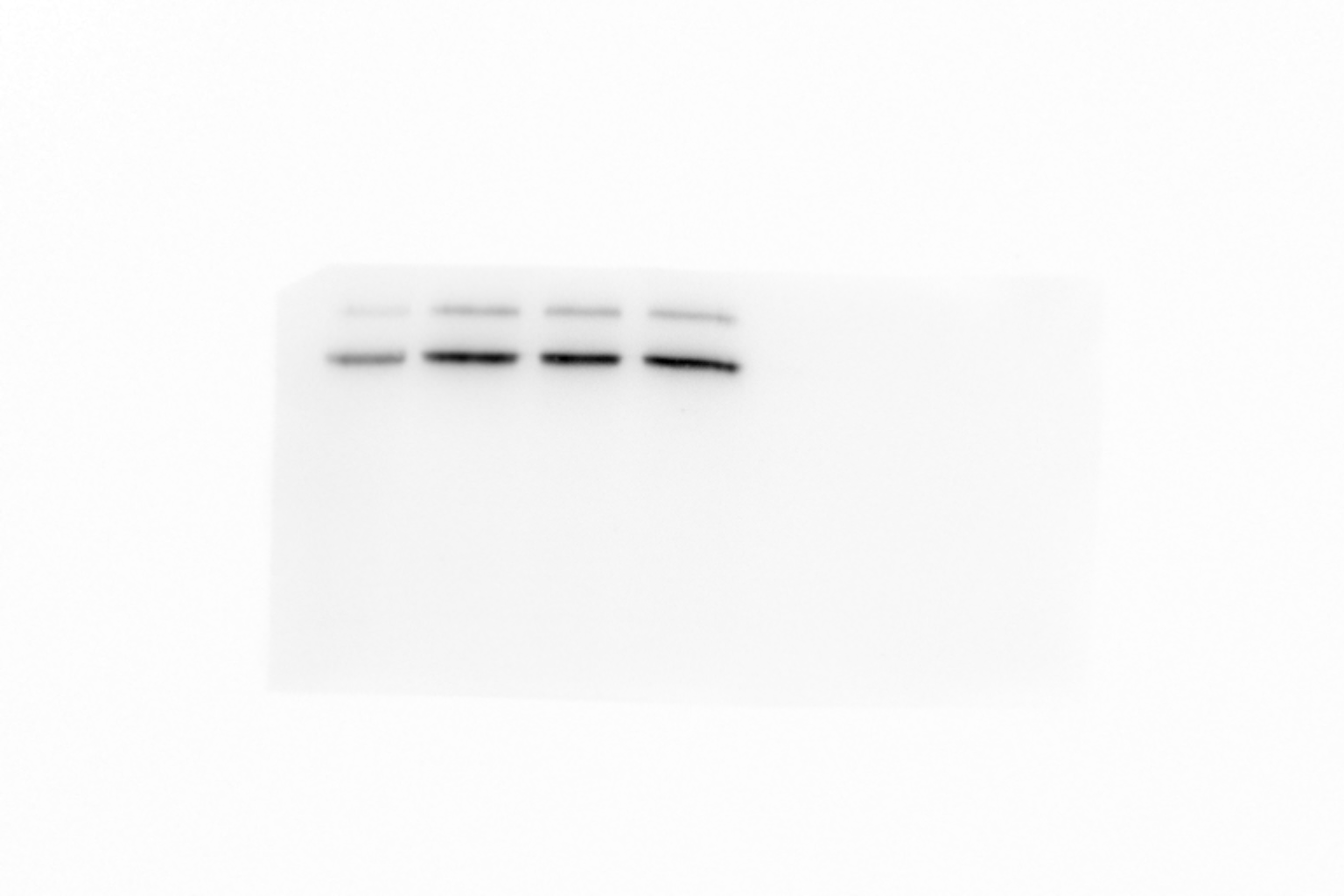

Supplement: Supplementary file 3 [file Data_Sheet_3.ZIP › 327021_images_3/Figure 8A P-S6K (T389).tif]

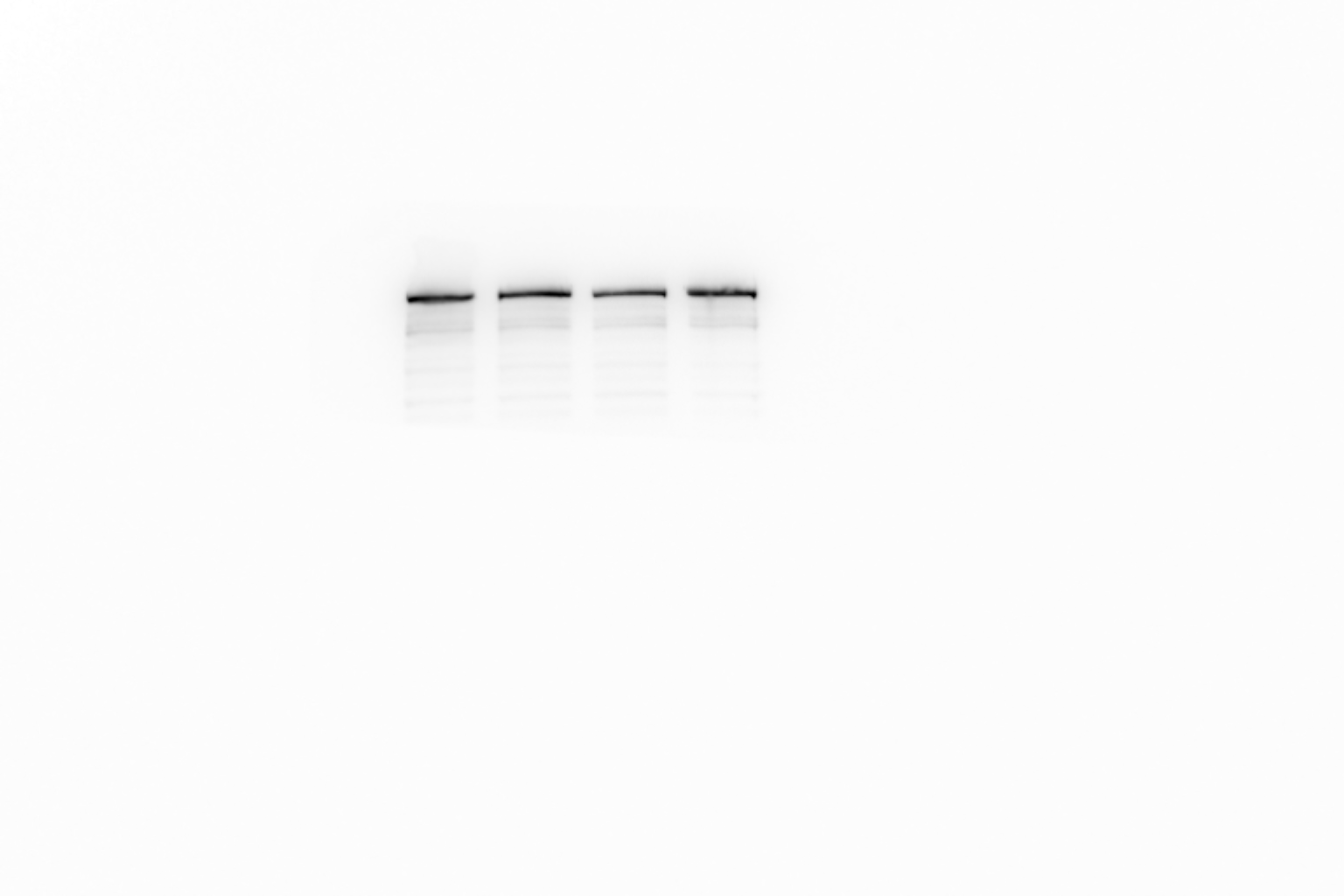

Supplement: Supplementary file 3 [file Data_Sheet_3.ZIP › 327021_images_3/Figure 8A mTOR.tif]

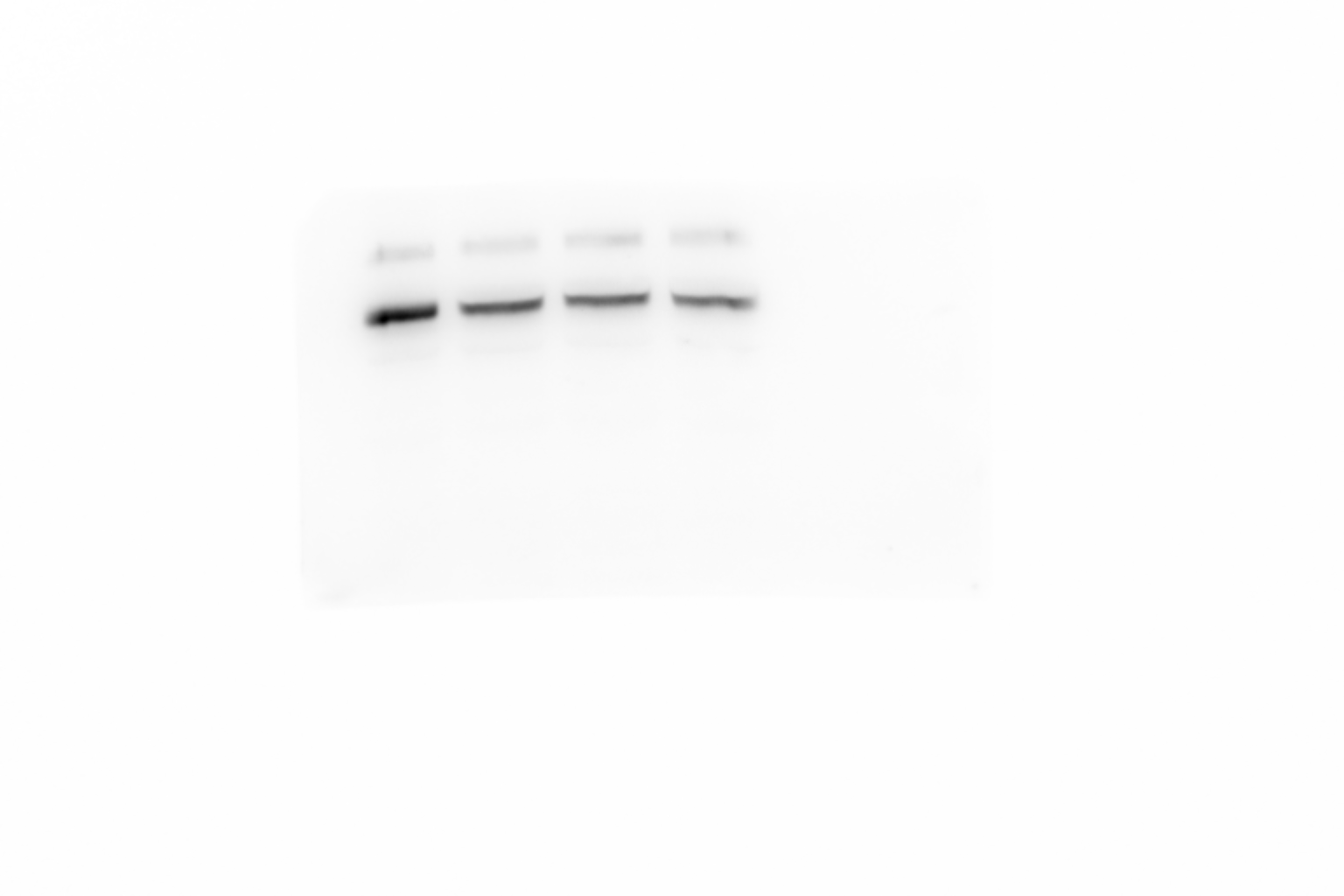

Supplement: Supplementary file 3 [file Data_Sheet_3.ZIP › 327021_images_3/Figure 8A S6K.tif]

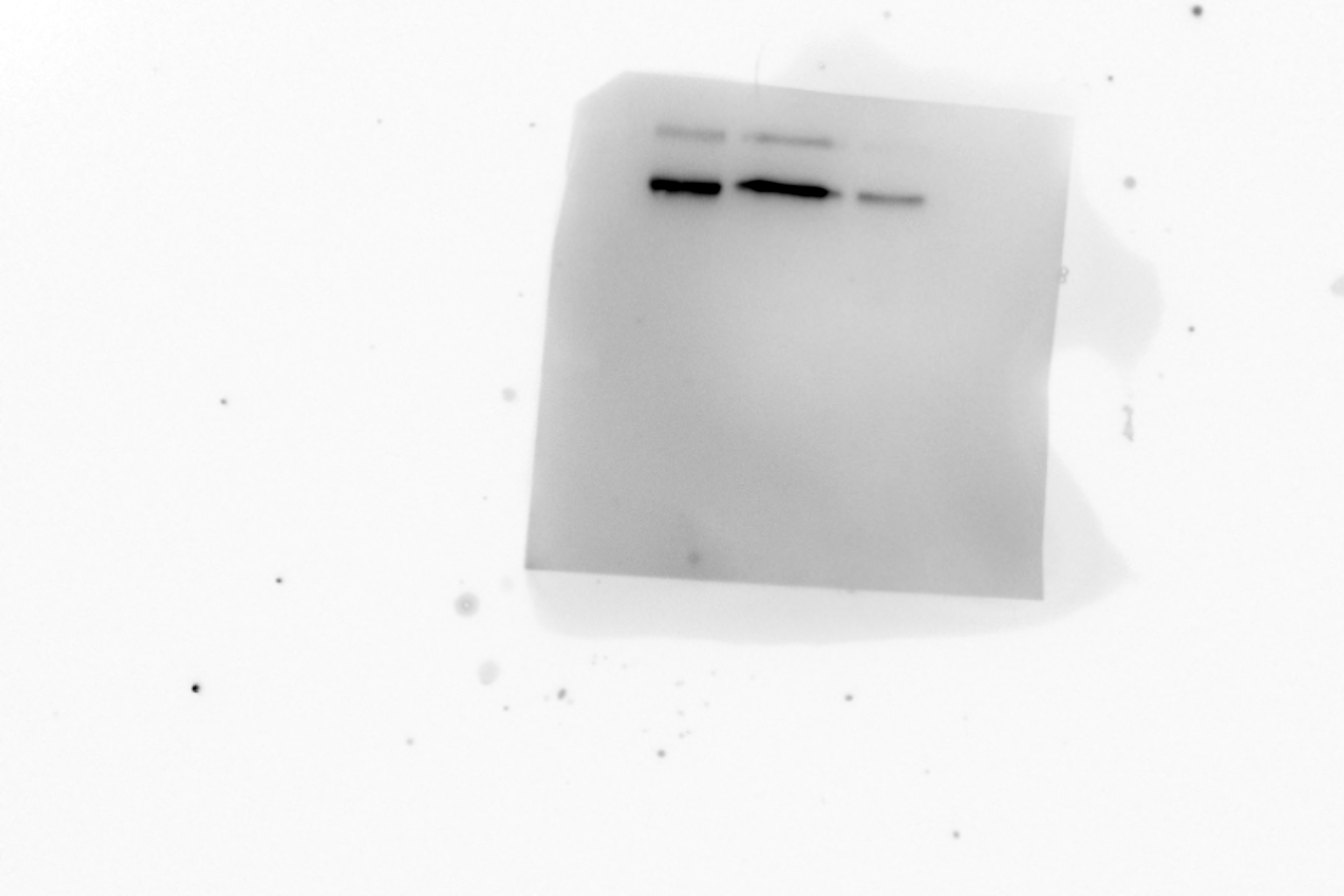

Supplement: Supplementary file 3 [file Data_Sheet_3.ZIP › 327021_images_3/Figure 8B P-S6K (T389).tif]

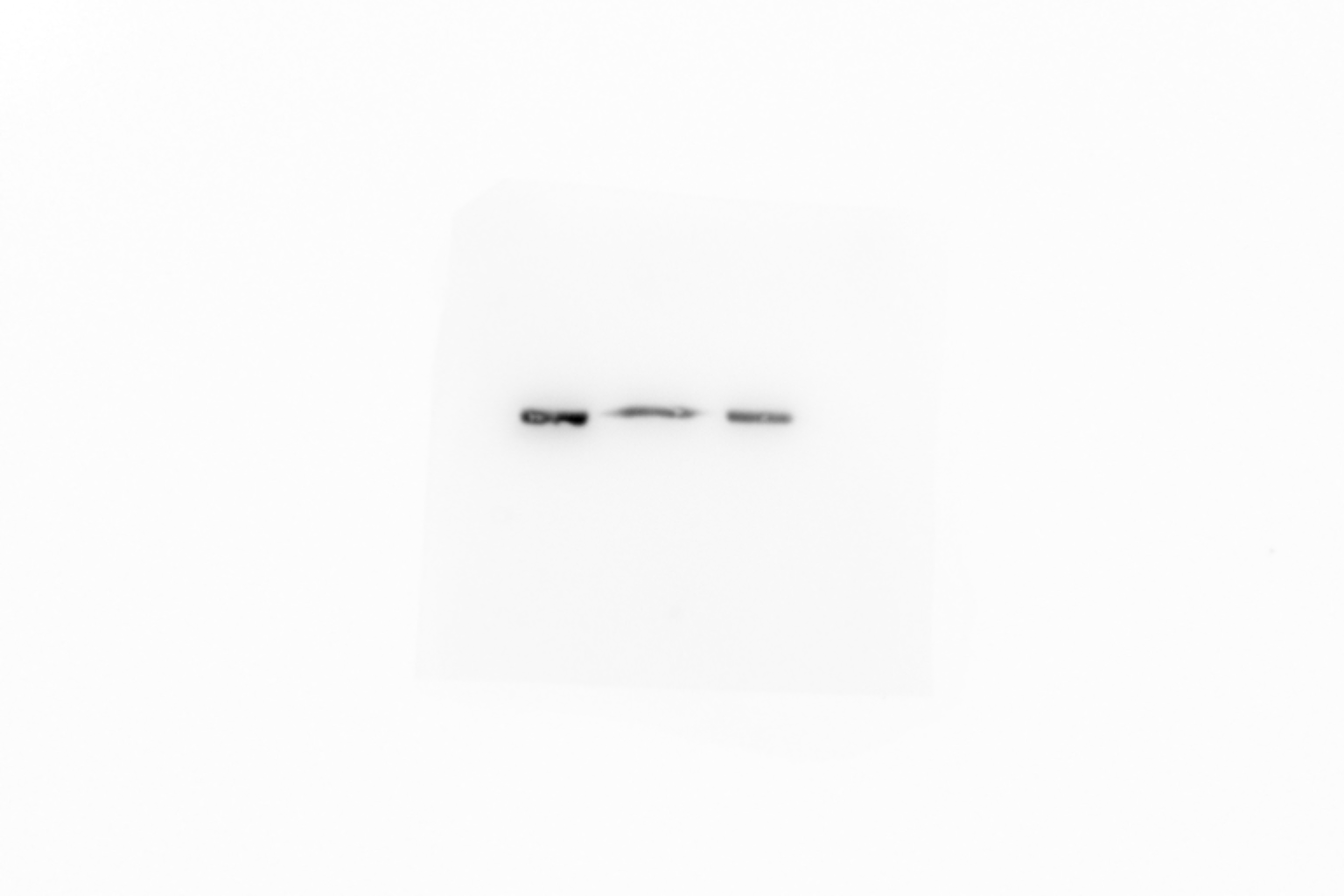

Supplement: Supplementary file 3 [file Data_Sheet_3.ZIP › 327021_images_3/Figure 8B S6K.tif]

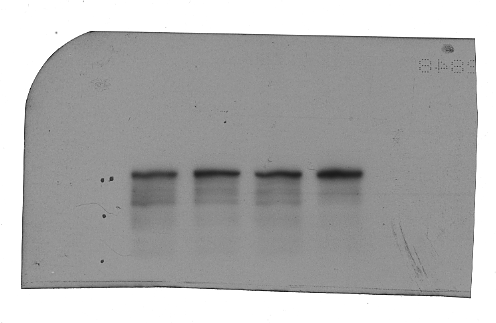

Supplement: Supplementary file 3 [file Data_Sheet_3.ZIP › 327021_images_3/Figure 8C mTOR.tif]

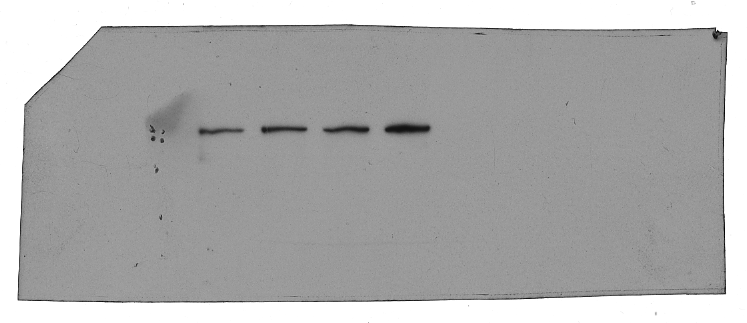

Supplement: Supplementary file 3 [file Data_Sheet_3.ZIP › 327021_images_3/Figure 8C P-mTOR (S2481).tif]

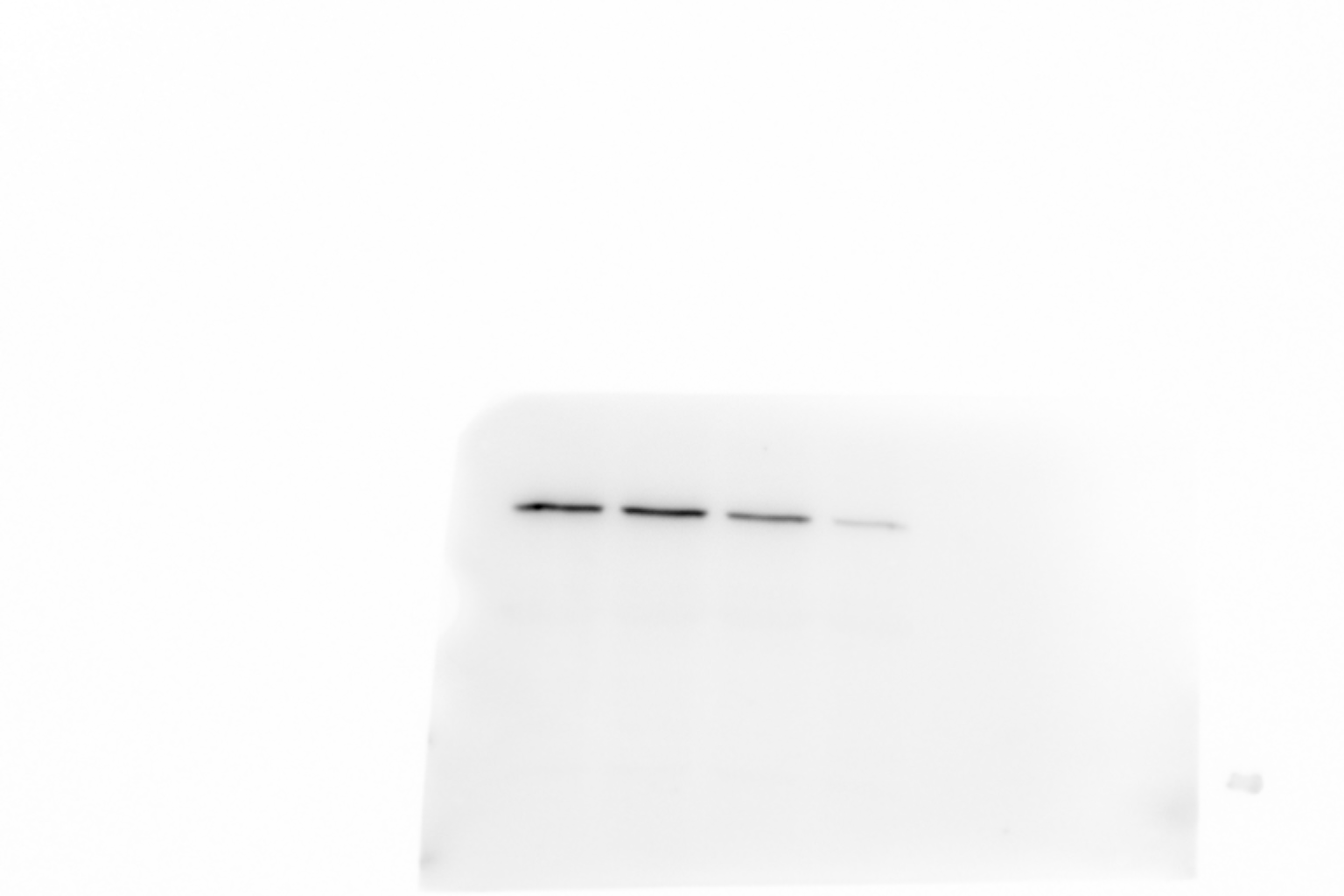

Supplement: Supplementary file 3 [file Data_Sheet_3.ZIP › 327021_images_3/Figure 8C P-PKB (S473).tif]

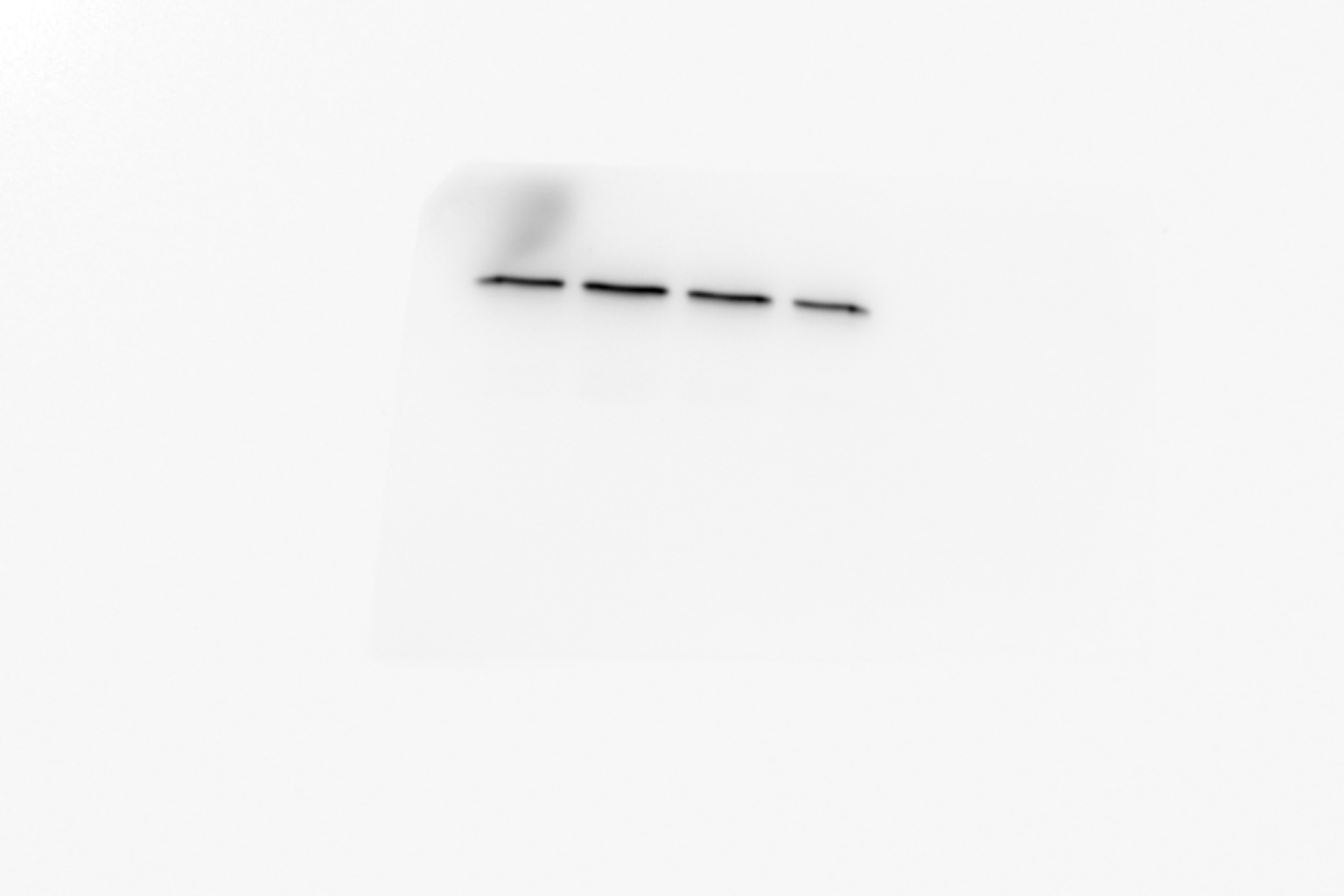

Supplement: Supplementary file 3 [file Data_Sheet_3.ZIP › 327021_images_3/Figure 8C PKB.tif]

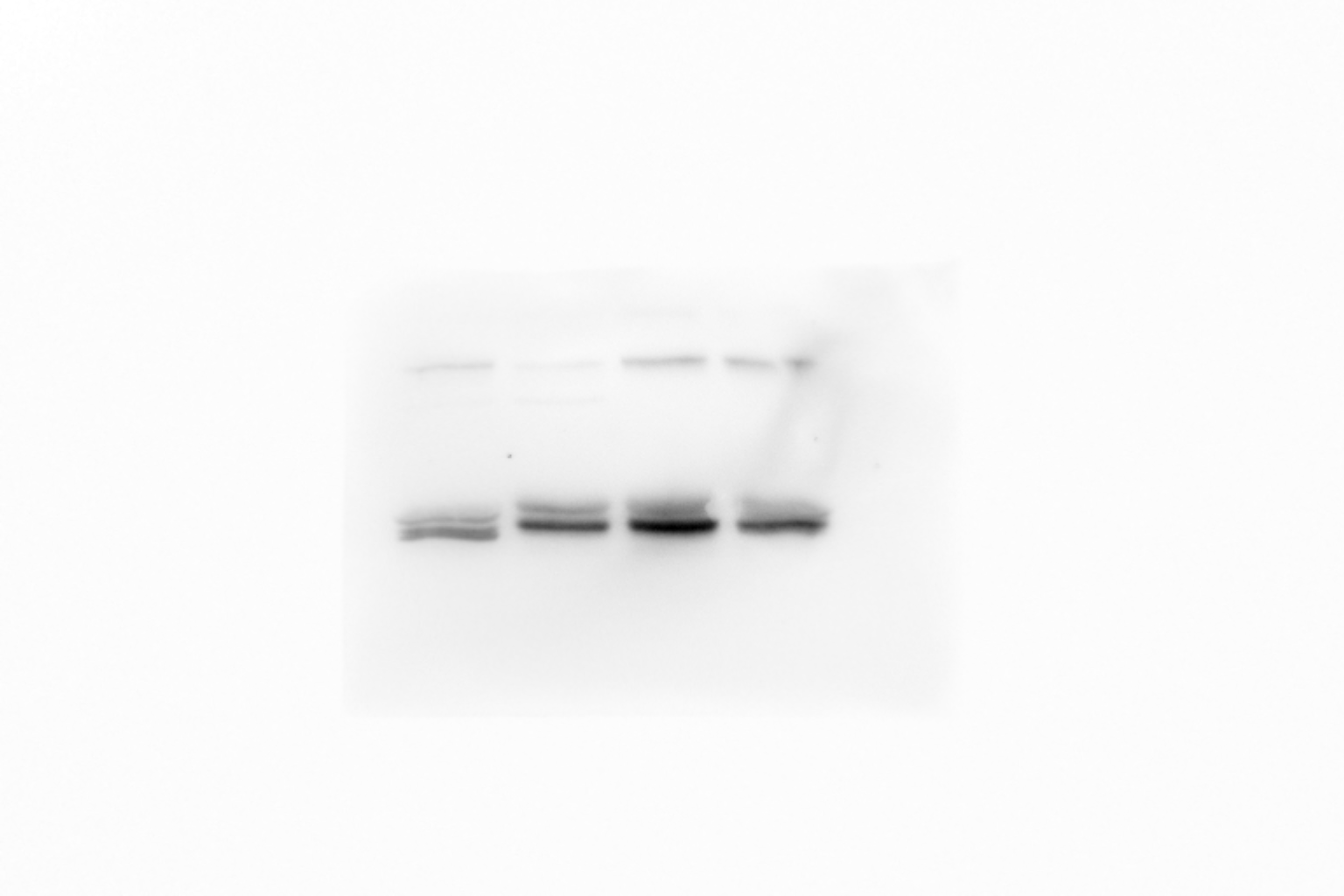

Supplement: Supplementary file 4 [file Data_Sheet_4.ZIP › 327021_images_4/Figure 8D ERK1_2.tif]

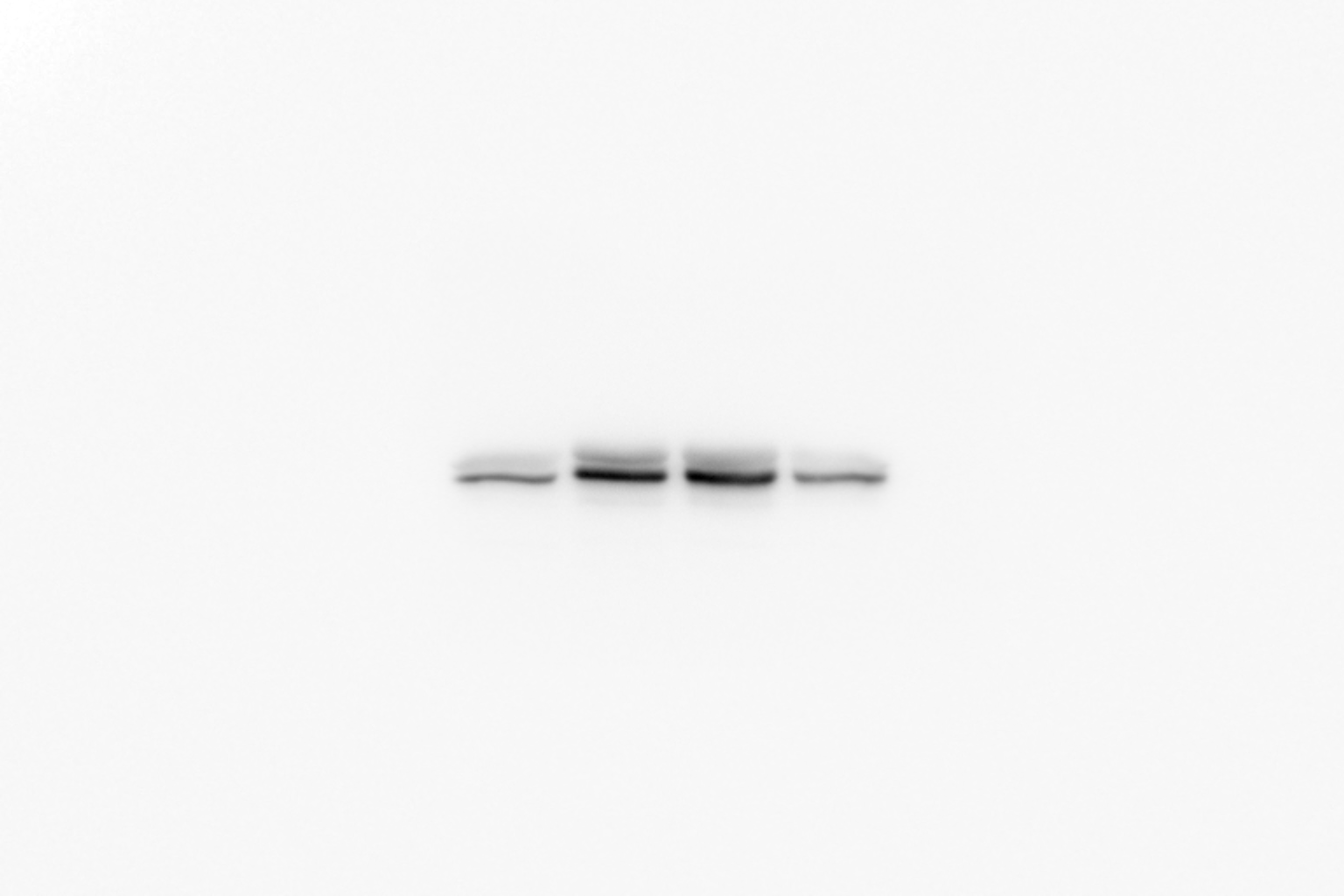

Supplement: Supplementary file 4 [file Data_Sheet_4.ZIP › 327021_images_4/Figure 8D P-ERK1_2 (T202_Y204).tif]

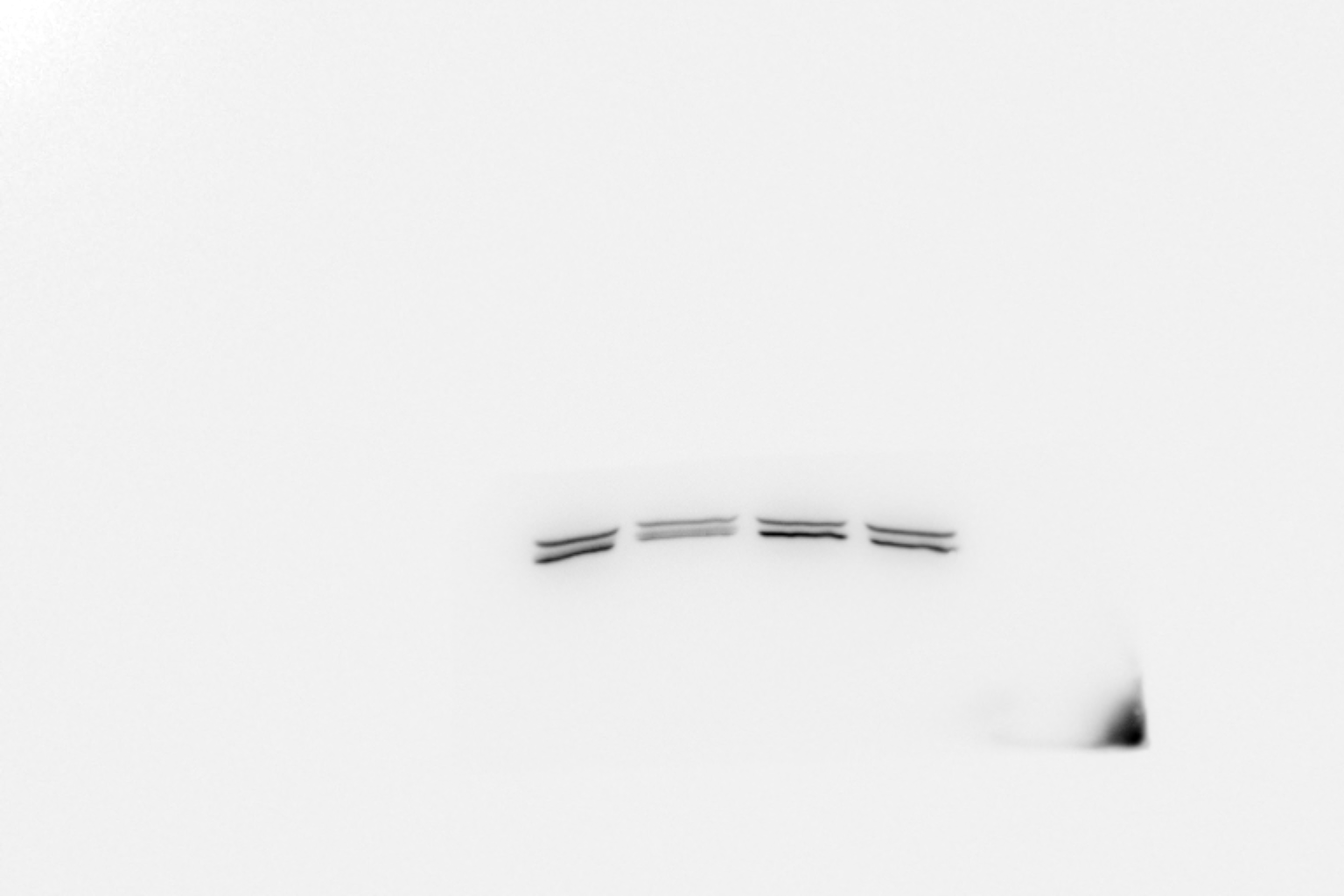

Supplement: Supplementary file 4 [file Data_Sheet_4.ZIP › 327021_images_4/Figure 8E ERK1_2.tif]

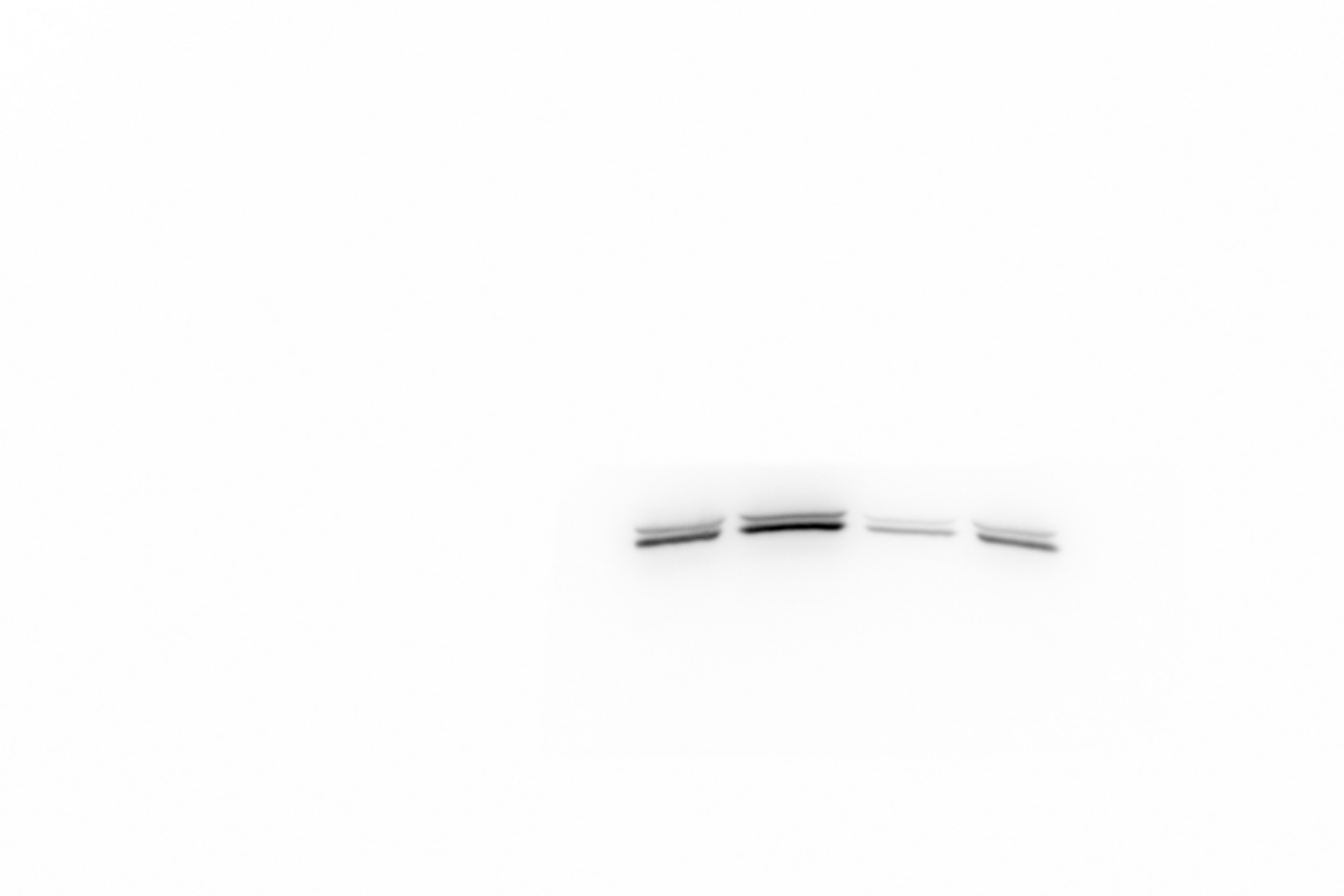

Supplement: Supplementary file 4 [file Data_Sheet_4.ZIP › 327021_images_4/Figure 8E P-ERK1_2 (T202_Y204).tif]

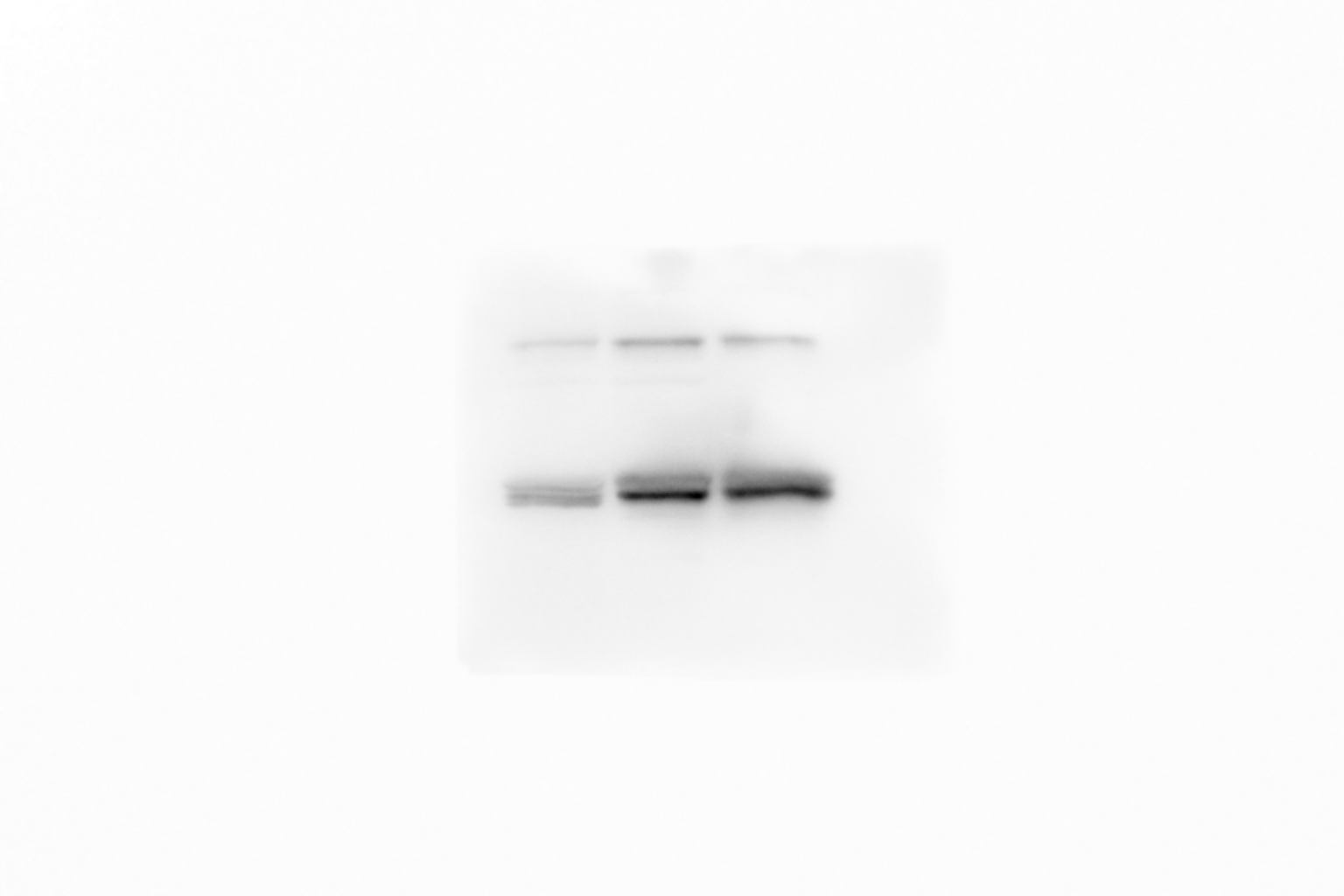

Supplement: Supplementary file 4 [file Data_Sheet_4.ZIP › 327021_images_4/Figure 8F ERK1_2.tif]

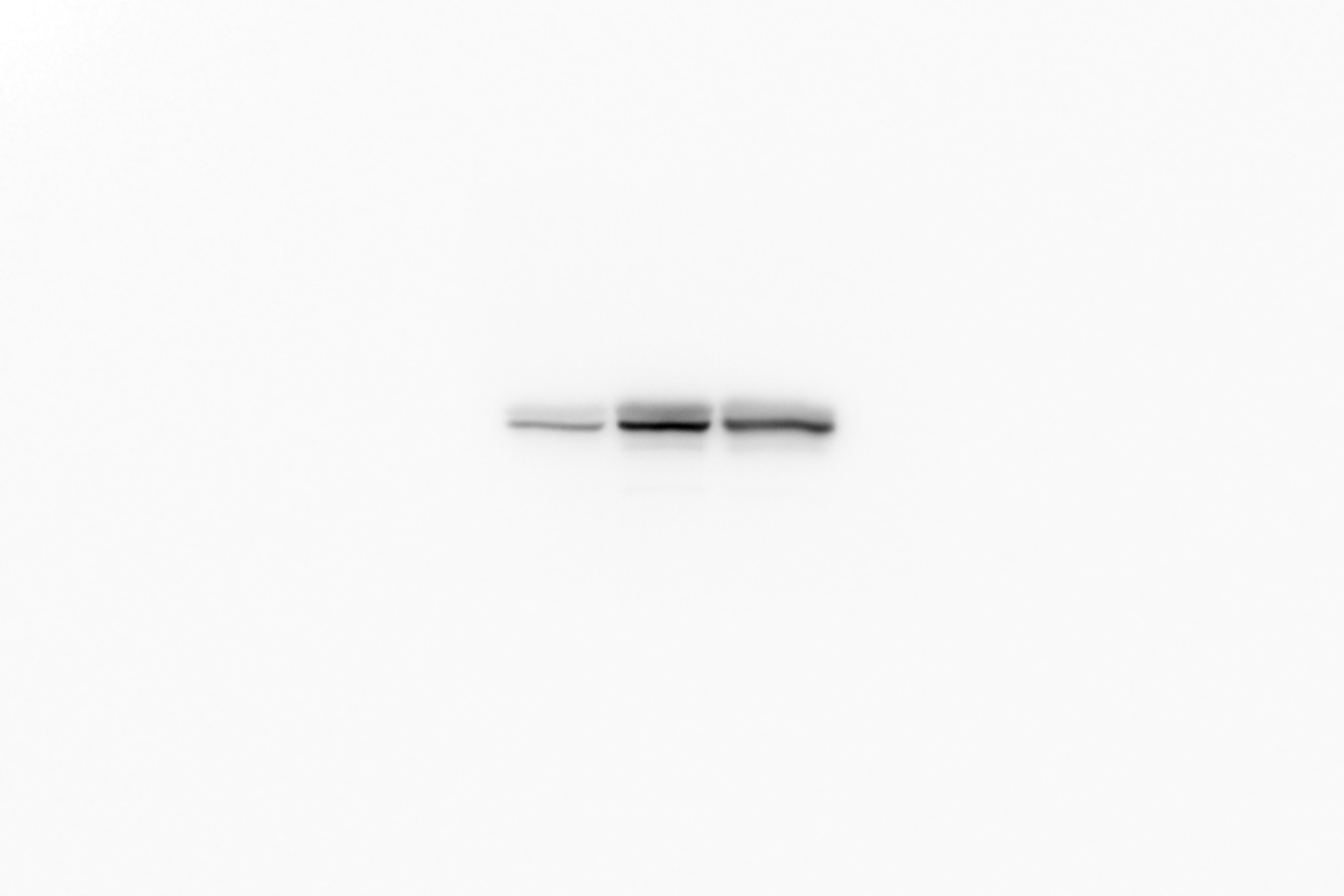

Supplement: Supplementary file 4 [file Data_Sheet_4.ZIP › 327021_images_4/Figure 8F P-ERK1_2 (T202_Y204).tif]

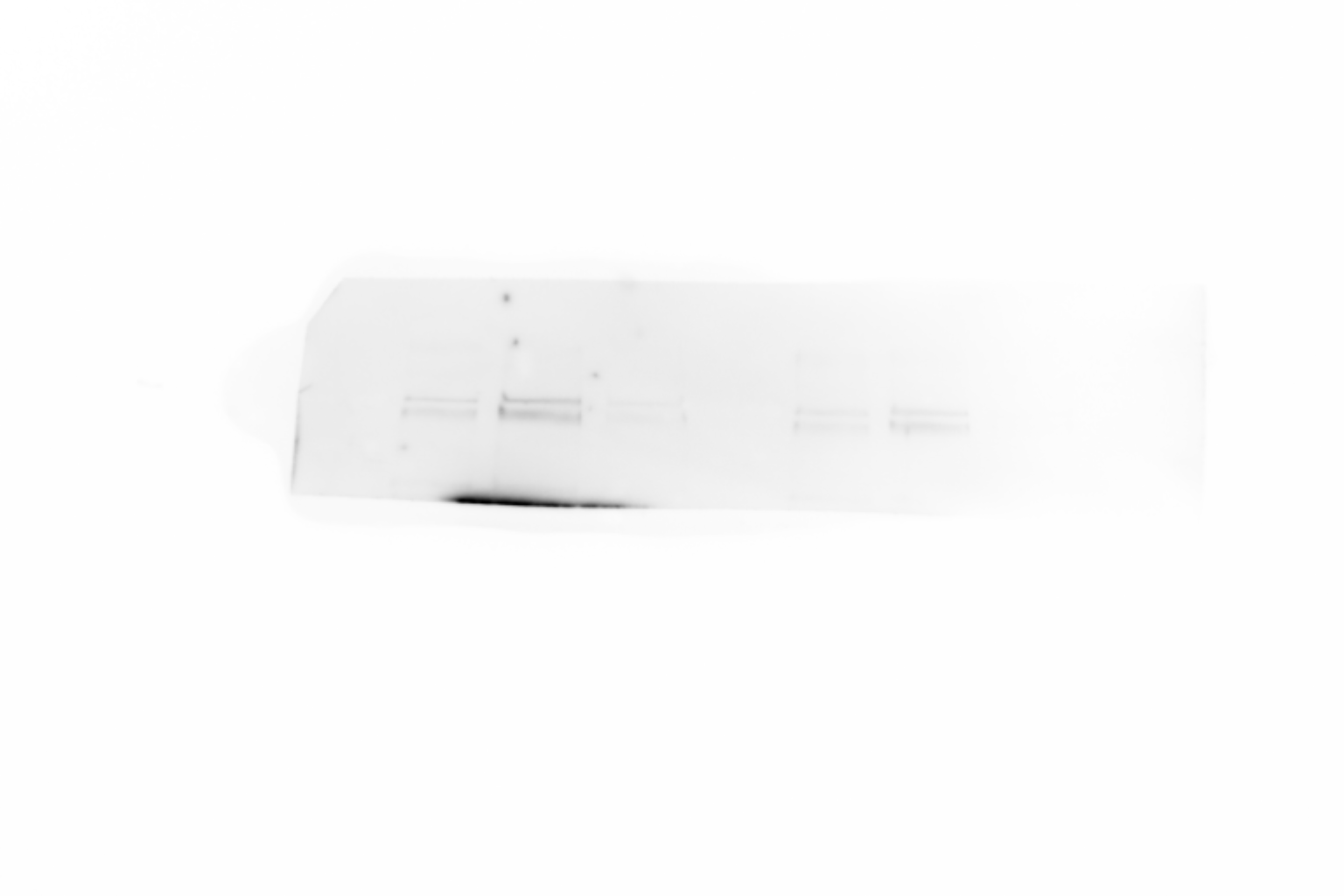

Supplement: Supplementary file 4 [file Data_Sheet_4.ZIP › 327021_images_4/Figure 8G P-TSC2 (S664).tif]

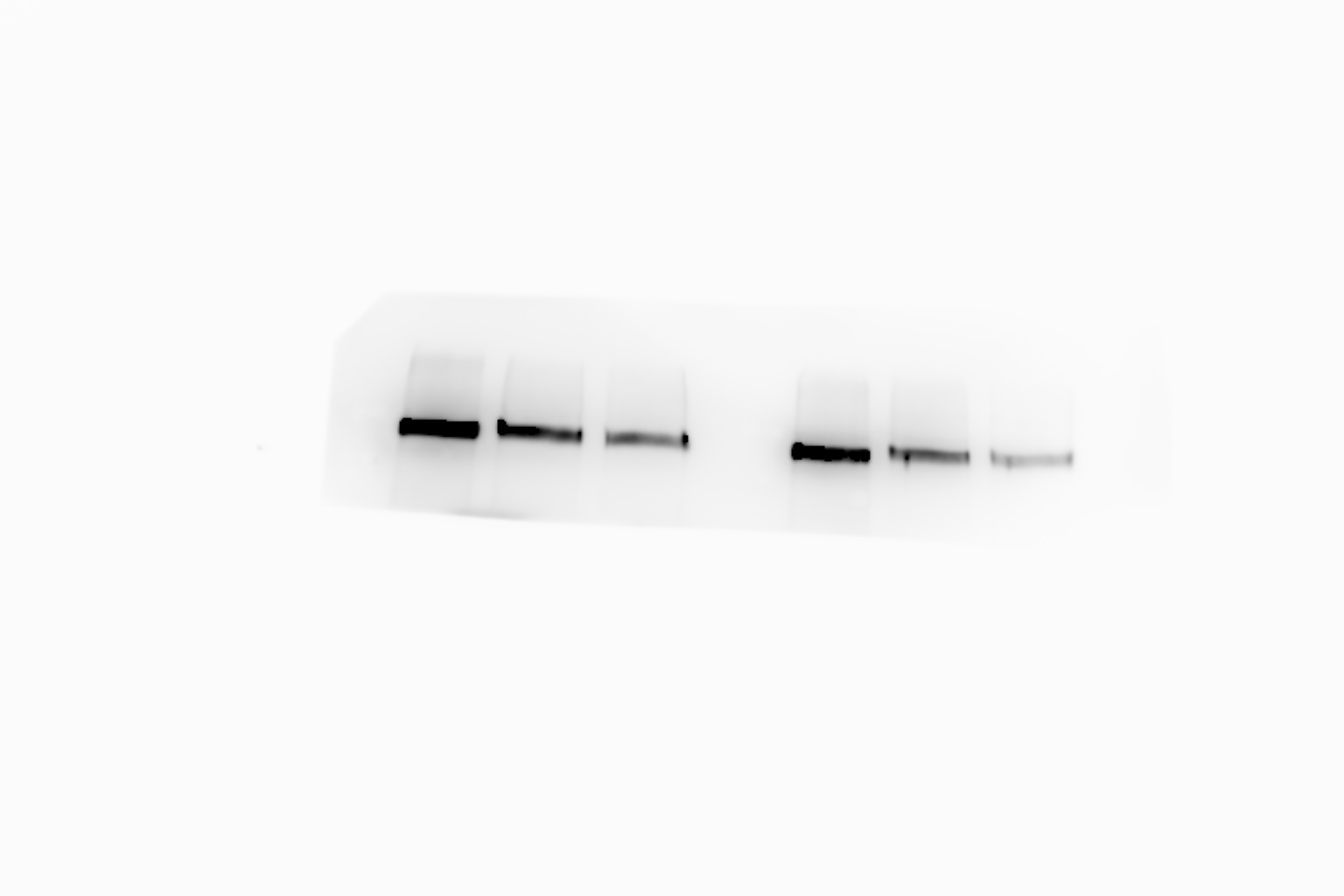

Supplement: Supplementary file 4 [file Data_Sheet_4.ZIP › 327021_images_4/Figure 8G TSC2.tif]

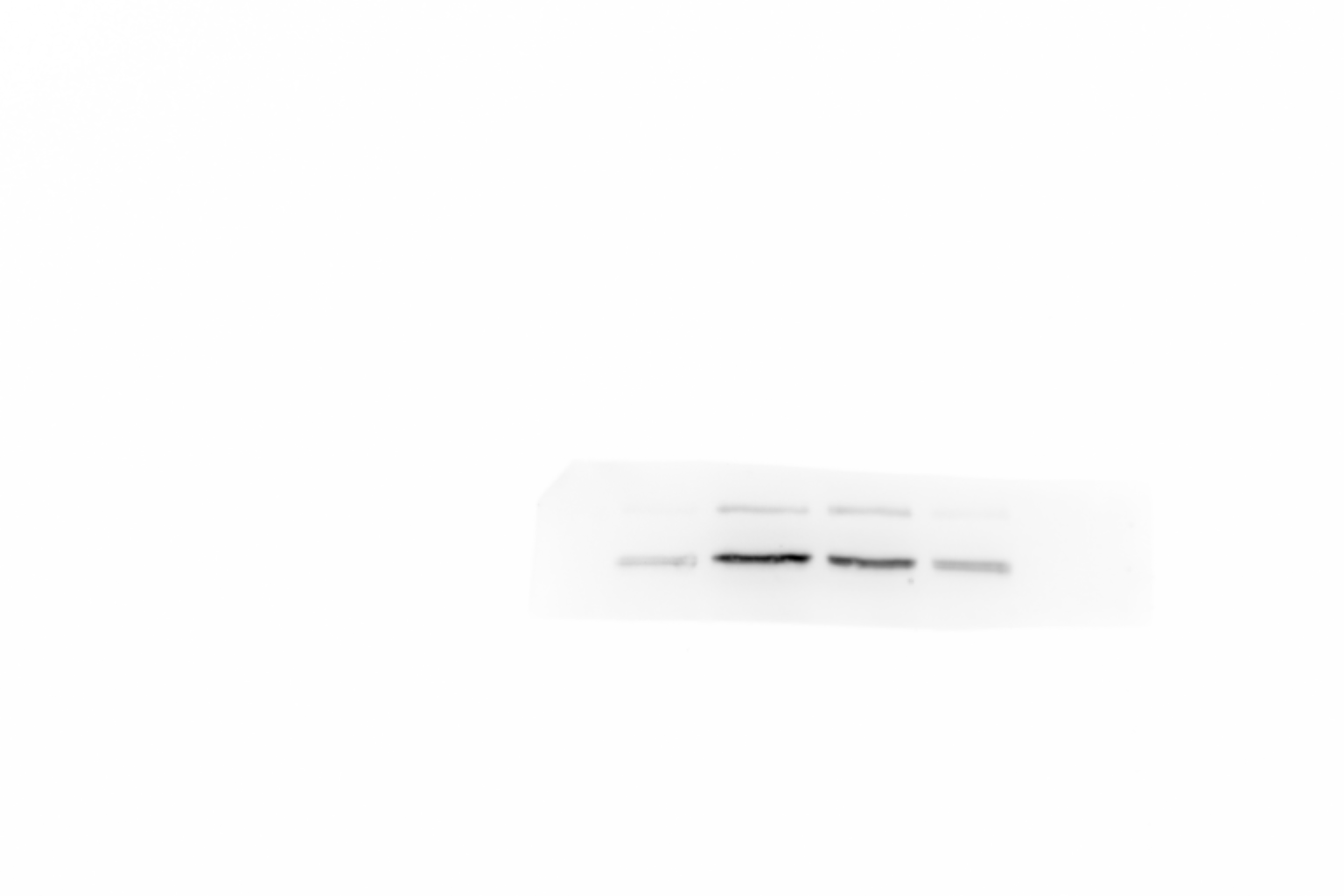

Supplement: Supplementary file 4 [file Data_Sheet_4.ZIP › 327021_images_4/Figure 8H P-S6K (T389).tif]

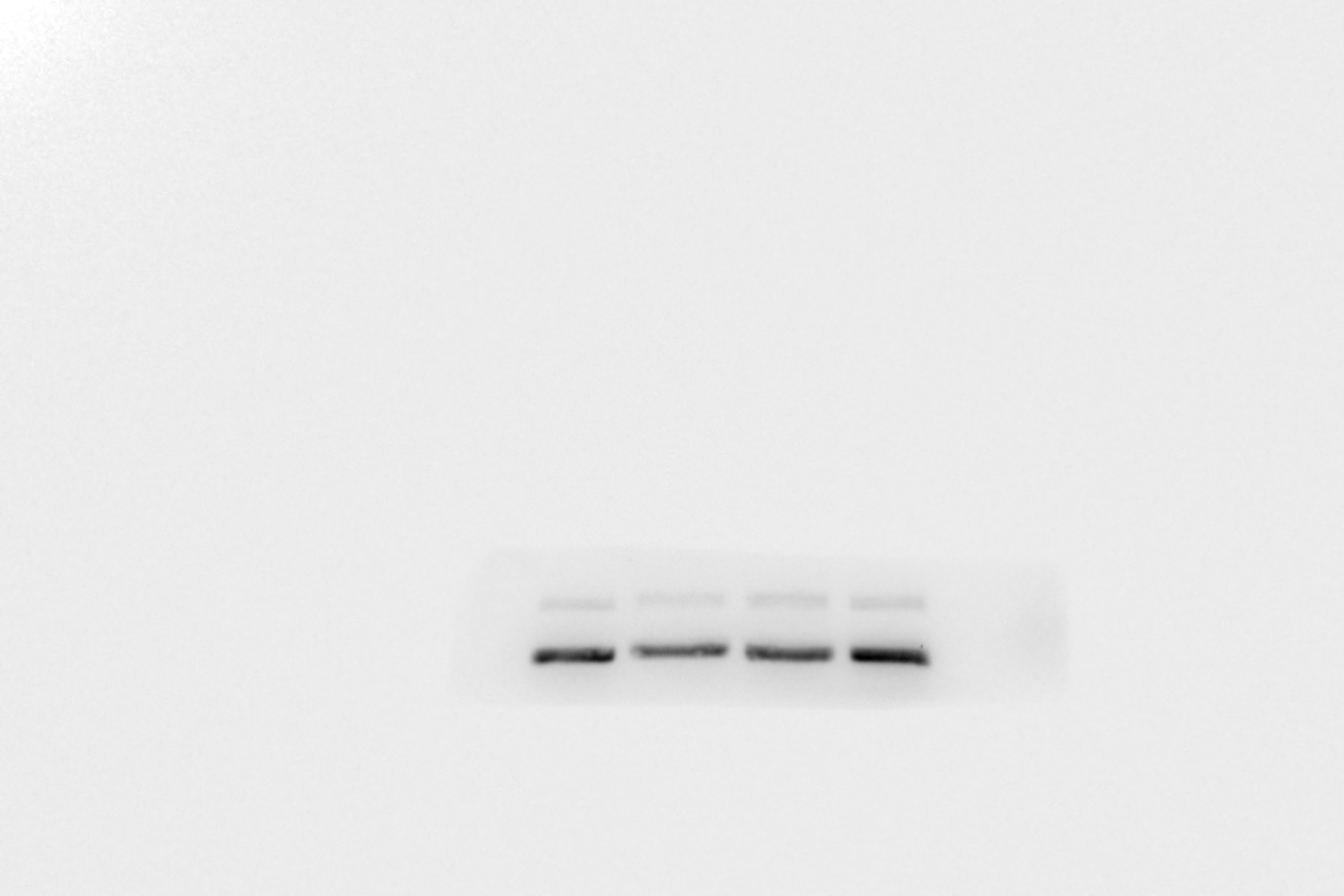

Supplement: Supplementary file 4 [file Data_Sheet_4.ZIP › 327021_images_4/Figure 8H S6K.tif]
